# Effects of Insulin Detemir and NPH Insulin on Body Weight and Appetite-Regulating Brain Regions in Human Type 1 Diabetes: A Randomized Controlled Trial

**DOI:** 10.1371/journal.pone.0094483

**Published:** 2014-04-16

**Authors:** Larissa W. van Golen, Dick J. Veltman, Richard G. IJzerman, Jan Berend Deijen, Annemieke C. Heijboer, Frederik Barkhof, Madeleine L. Drent, Michaela Diamant

**Affiliations:** 1 Diabetes Center/Department of Internal Medicine, VU University Medical Center, Amsterdam, The Netherlands; 2 Department of Psychiatry, VU University Medical Center, Amsterdam, The Netherlands; 3 Department of Clinical Neuropsychology, VU University, Amsterdam, The Netherlands; 4 Department of Clinical Chemistry, VU University Medical Center, Amsterdam, The Netherlands; 5 Department of Radiology & Nuclear Medicine, VU University Medical Center, Amsterdam, The Netherlands; 6 Department of Internal Medicine/Endocrine Section, VU University Medical Center, Amsterdam, The Netherlands; Postgraduate Medical Institute & Hull York Medical School, University of Hull, United Kingdom

## Abstract

Studies in rodents have demonstrated that insulin in the central nervous system induces satiety. In humans, these effects are less well established. Insulin detemir is a basal insulin analog that causes less weight gain than other basal insulin formulations, including the current standard intermediate-long acting Neutral Protamine Hagedorn (NPH) insulin. Due to its structural modifications, which render the molecule more lipophilic, it was proposed that insulin detemir enters the brain more readily than other insulins. The aim of this study was to investigate whether insulin detemir treatment differentially modifies brain activation in response to food stimuli as compared to NPH insulin. In addition, cerebral spinal fluid (CSF) insulin levels were measured after both treatments. Brain responses to viewing food and non-food pictures were measured using functional Magnetic Resonance Imaging in 32 type 1 diabetic patients, after each of two 12-week treatment periods with insulin detemir and NPH insulin, respectively, both combined with prandial insulin aspart. CSF insulin levels were determined in a subgroup. Insulin detemir decreased body weight by 0.8 kg and NPH insulin increased weight by 0.5 kg (*p* = 0.02 for difference), while both treatments resulted in similar glycemic control. After treatment with insulin detemir, as compared to NPH insulin, brain activation was significantly lower in bilateral insula in response to visual food stimuli, compared to NPH (*p* = 0.02 for right and *p* = 0.05 for left insula). Also, CSF insulin levels were higher compared to those with NPH insulin treatment (*p* = 0.003). Our findings support the hypothesis that in type 1 diabetic patients, the weight sparing effect of insulin detemir may be mediated by its enhanced action on the central nervous system, resulting in blunted activation in bilateral insula, an appetite-regulating brain region, in response to food stimuli.

**Trial Registration:**

ClinicalTrials.gov NCT00626080.

## Introduction

In addition to its blood glucose lowering peripheral effects, insulin acts within the central nervous system (CNS) to regulate eating behavior and energy balance [1]. Animal studies have shown that disrupted intracerebral insulin signaling causes weight gain and that intracerebroventricular insulin administration reduces food intake and body weight [2], [3]. In humans, central insulin action was studied using intranasal insulin [4]; acute intranasal insulin promoted satiety and reduced snacking in the postprandial state in women [5] and resulted in weight loss in the longer term in men [6]. However, it is not clear whether effects of intranasal insulin are completely independent of insulin's peripheral action. Besides, intranasal insulin is not clinically available.

Patients with type 1 diabetes (T1DM) require treatment with multiple subcutaneous insulin injections daily. Insulin detemir (ID) is a more recently developed basal insulin analog, that has been associated with body weight loss compared to weight gain after treatment with other basal insulin formulations [7]. ID differs from human insulin in that threonine at position B30 has been removed and that lysine at B29 has been acylated with myristic acid, a 14-carbon fatty acid. This fatty-acid moiety stabilizes ID self-association and enables the binding to albumin, which gives ID its long-acting properties [8]. It has been hypothesized [9] that due to the fatty acid moiety, ID more easily enters the brain, thereby potentially promoting satiety in relevant CNS regions and reducing appetite, food intake and body weight. Accordingly, ID may have stronger effects in modulating brain functions than other long-acting insulin formulations: ID, administered intravenously, enhanced cortical activity compared to human insulin (as measured by electroencephalography, EEG and magnetoencephalography, MEG) and decreased food intake in both preclinical [10] and clinical studies [11], [12]. However, the effects of ID on appetite regulating brain regions during food stimuli have not been studied and no data are available regarding insulin concentrations in human cerebrospinal fluid (CSF) during insulin treatment and its relation to insulin's central actions.

In previous functional MRI (fMRI) studies, it was shown that obese subjects show hyperactivation to food pictures in brain networks linked to motivation, reward and cognitive control [13], [14] and that fMRI hyperactivation to high-calorie food pictures predicts weight gain [15]. In the present study, fMRI was used to test the hypothesis that treatment with ID modifies activation in appetite regulating brain regions in response to visual food stimuli compared to regular treatment with Neutral Protamine Hagedorn insulin (NPH). The study was performed in T1DM patients since these patients are insulin-deficient, allowing the assessment of effects attributable to exogenous insulin per se. Indeed, differences were observed in brain activation between treatment with ID and NPH.

## Materials and Methods

The protocol for this trial and supporting CONSORT checklist are available as supporting information; see Checklist S1 and Protocol S1.

### Ethics statement

The study was approved by the Medical Ethics Review Committee of the VU University Medical Center (VUMC) and the Central Committee on Research involving Human Subjects; the study was conducted according to the Declaration of Helsinki. All participants gave written informed consent before inclusion in the study.

### Participants

**Figure 1:**
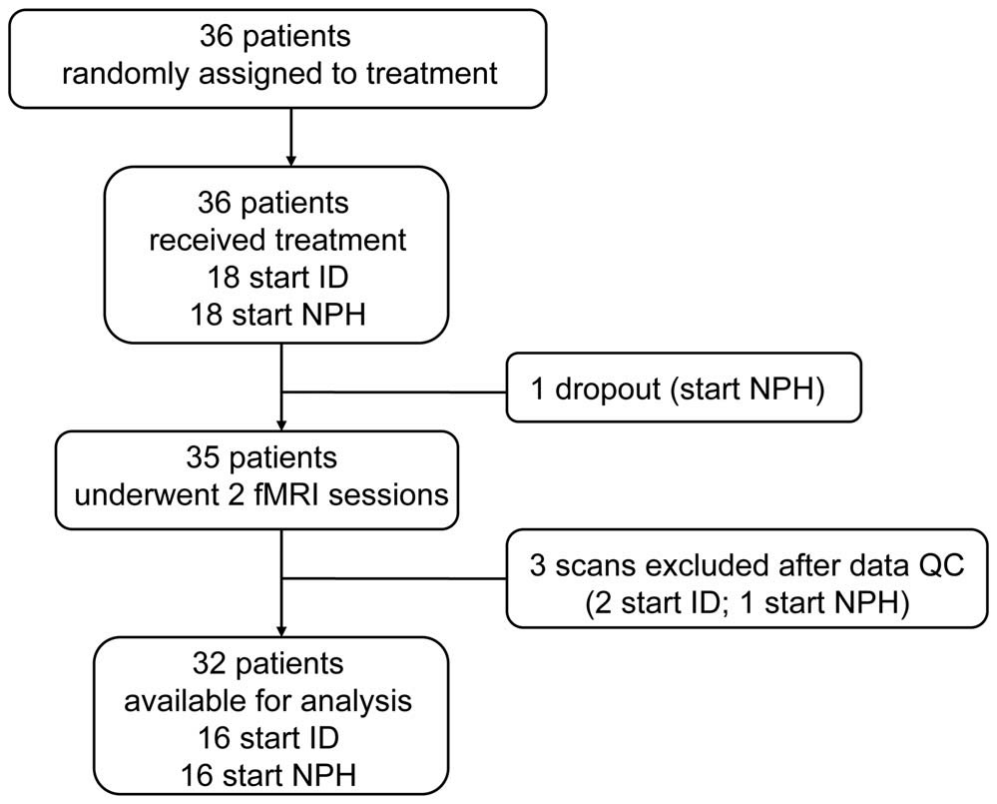
Participant flow diagram. ID, insulin detemir; NPH, Neutral Protamine Hagedorn insulin; QC, quality control.

From January 2009 until May 2011, 36 T1DM men were included in the study (Figure 1). The last follow-up visit was on 13 December 2011. Patients (aged 18–60 years; BMI 18–35 kg/m^2^) were recruited from the outpatient clinic, from neighboring hospitals and through advertisements in local newspapers. All subjects underwent a screening visit, consisting of medical history taking, physical examination and fasting blood and urine analyses. Exclusion criteria were a history of cardiovascular, renal and liver disease, severe head trauma, neurological or psychiatric disorders, endocrine diseases not well-controlled for the last three months, inability to undergo MRI scanning, substance abuse or the use of anticoagulants, oral steroids or any centrally acting agent. In addition diabetes duration less than 1 year, glycated hemoglobin HbA1c>8.5% (69 mmol/mol), a history of or current proliferative retinopathy, a history of recurrent severe hypoglycemia (defined as an episode that requires external assistance to aid recovery), or a medical history of hypoglycemia unawareness were exclusion criteria. Any current insulin therapy was allowed, as long as patients were willing to switch to the basal-bolus study insulin treatment.

### Protocol and intervention

**Figure 2:**
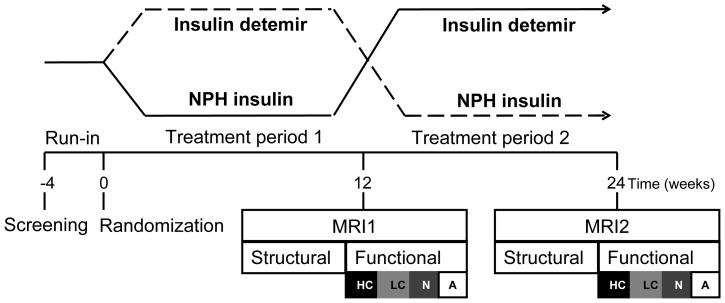
Study design. After a run-in period, patients were randomly assigned to treatment with either insulin detemir or NPH insulin. After each treatment period an fMRI-scan was acquired. During the fMRI, pictures were shown of four categories in random order (HC, high calorie food; LC, low calorie food; N, non-food; A, arrow).

This study was part of the INcEREBRO study (ClinicalTrials.gov, NCT00626080) and was conducted according to a cross-over design (Figure 2). After a run-in period of at least four weeks, during which their current insulin therapy was optimized, patients were randomly assigned to start with either ID or NPH in the evening, in combination with insulin aspart at mealtimes. Randomization (block design) was conducted by the Trial Pharmacy of the VUMC and the assigned treatments were concealed by envelopes. Since NPH and ID can be visually distinguished as NPH is a cloudy solution that needs to be mixed before injection, while the ID preparation is available as a clear solution, blinding of insulin treatment was not possible. After each 12-week treatment-period patients underwent an fMRI. Also, immediately after the MRI measurement, a lumbar puncture (LP) was performed to obtain CSF for measurement of insulin levels. Undergoing an LP was made optional, and was performed only in a subgroup of patients who signed an additional informed consent.

All participants received detailed information about the scanning procedure, but were not aware of the food-related nature of the experiment, to avoid anticipation. They were instructed to refrain from eating, alcohol or coffee from 10 PM the night before scanning. Patients were instructed to inject their basal insulin and, if possible, not to use any insulin aspart after their dinnertime injection. They were instructed to measure their blood glucose at home and send the value via text message to the research physician in order to receive further instructions if necessary; they arrived at the hospital at 7.15 AM. Upon arrival, in all participants blood was drawn and blood glucose level was checked and corrected if necessary (when glucose was <4 mmol/L and/or falling [relative to early morning self-monitored glucose levels], glucose was injected intravenously; no insulin aspart was injected); just before and immediately after the MRI glucose was measured as well.

### Questionnaires

Before and directly after scanning, patients completed questionnaires rating hunger, fullness, appetite, prospective consumption, desire to eat and thoughts of eating on a 10-point Visual Analog Scale as well as a shortened version of the profile of mood state (POMS) before and after scanning [16]. Furthermore, patients completed the DTSQ (Diabetes Treatment Satisfaction Questionnaire) [17] and the Dutch Eating Behaviour Questionnaire (DEBQ) [18] once.

### fMRI paradigm

The fMRI task consisted of the presentation of four types of pictures, showing 1) high calorie food, 2) low calorie food, 3) non-food objects and 4) arrows, adapted from [19]. Pictures were assembled both from commercial stock photography websites and from self-made pictures. All pictures were full-color, high resolution photographs, showing the four types of pictures either in an indoor or an outdoor background, resulting in eight different combinations, i.e. high calorie x indoor, high calorie x outdoor, low calorie x indoor, low calorie x outdoor, non-food x indoor, non-food x outdoor, arrow left and arrow right (presented via Eprime software [19]).

Participants were placed in a supine position in the scanner with their head placed in a passive restraint (pads around the head) to minimize motion. fMRI was performed while the subjects were presented the above described visual stimuli using a projector and screen system viewed through a mirror. Before image acquisition started, participants performed a test task to ensure that the task was understood properly. The real task consisted of eleven pseudo-randomized blocks, each block containing three to eight different pictures of the same category. In order to control for attention lapses, participants were requested to make indoor-outdoor judgments using an MRI-compatible response box. Each block was followed by one to three scrambled arrows (left or right), to which participants had to respond by indicating the direction of the arrow. Each stimulus (picture and arrow) was shown for four seconds, followed by an inter-stimulus interval (fixation cross) of 1.5 to 2.5 seconds. The task lasted approximately eight minutes. Since each patient was scanned twice, two different versions of the task, with equal design, were presented. The order of scanning versions was randomized across subjects, to prevent order effects.

### Image acquisition and analysis

MRI data were acquired on a 3.0 T GE SignaHDxt scanner (General Electric, Milwaukee, Wisconsin, USA). A 3-D structural MRI was obtained using a T1-weighted FSPGR (fast Spoiled Gradient echo) sequence with the following parameters: TR (Repetition Time) = 7.8 ms; TI (Inversion Time) = 450 ms; TE (Echo Time) = 3.0 ms; FA (flip angle) = 12^o^; voxel size  = 1×0.94×0.94 mm. fMRI data were acquired using echo planar imaging (EPI) T2* BOLD (Blood Oxygen Level Dependent) pulse-sequence (TR = 2160 ms, TE = 30 ms, matrix 64×64, 211 mm^2^ field of view, 40 slices angled parallel to the planum sphenoidale, 3 mm thickness, 0 mm gap).

Image data were analyzed using SPM8 software (Wellcome Trust Center for Neuroimaging, UK). The origin of each MR volume was aligned to the anterior commissure. Series were corrected for differences in slice acquisition times and were realigned to the first volume. T1-coregistered volumes were normalized to Montreal Neurological Institute (MNI) space, resliced to 3×3×3 mm voxels and spatially smoothed using an 8mmfull width at half maximum Gaussian kernel. After high-pass filtering (cut-off 128 s), functional scans were analyzed in the context of the general linear model using box-car functions convolved with a canonical hemodynamic response function to model responses during each block. For each subject, activation contrasts were computed. Contrast images derived from first-level (within subject) analyses were entered into second level (between subject) (random effects) analyses. Paired t-tests were performed for treatment effect, masked with main effect of contrast. A priori regions of interest (ROIs) were determined based on areas activated by cues of food rewards in previous studies (i.e., insula, striatum, orbitofrontal cortex). When ROIs corresponded to discrete anatomical structures, small volume correction, using 5 mm (for anterior cingulate cortex, ACC) or 10 mm (for insula) radius spheres [20], was used for multiple comparisons applied at *p*<.05.

### Biochemical analyses

Capillary blood glucose was measured using a calibrated blood glucose meter (OneTouch ultra easy, LifeScan, Inc. Milpitas, CA, USA). HbA1c was measured in the VUMC central laboratory, by cation-exchange chromatography (reference value: 4.3–6.1% (23–43 mmol/mol); Menarini Diagnostics, Florence, Italy). Serum insulin concentrations were quantified using an immunometric assay. (Centaur, Siemens Diagnostics, Deerfield, IL). Cross-reactivity was calculated using the slope of the Passing &Bablok analysis. Cross-reactivity was 100% for NPH and 120% for ID. CSF samples were centrifuged at 2750 RPM for 10 min at 4°C, within one hour after collection. A small amount of CSF was used for routine analysis, including total cell count, total protein and glucose. CSF was aliquoted in polypropylene tubes of 0.5 mL and stored at −80°C until further analysis [21]. CSF insulin levels were measured using an ultrasensitive RIA (EMD Millipore, Billerica, MA).Cross-reactivity of NPH and ID were 140% and 20%, respectively. Cholesterol (total, HDL, triglycerides) was measured using an enzymatic calorimetric assay (Modular P, Roche Diagnostics, Basel, Switzerland). Urine microalbumin was quantified using immunonephelometry (Immage 800, Beckman Coulter, Brea, CA).

### Statistical analysis

Clinical data are expressed as mean ± SD, skewed data and ordinal values are expressed as median and inter-quartile range. Treatment effects were analyzed using repeated measure analysis, or Wilcoxon signed-rank test. Univariate correlations (Pearson's *r* or Spearman's *r*) were used to examine associations. Analyses were run on SPSS (SPSS Inc., Chicago, IL), version 20. *p*<.05 was considered statistically significant.

The effects of two different insulin regimens on fMRI-measured BOLD signal have not been investigated before. Other fMRI studies using a parallel group or paired designs to compare two groups or post-pre intervention, respectively, required 6-26 individuals to show meaningful results [15], [22], [23]. Based on these studies and our premise that the difference on brain activation between the two insulin regimens would be modest (15%, standard deviation 20%), and assuming a power of .8 and a two-sided .05 significance level, we calculated that in a cross-over study design 30 patients would be needed.

## Results

### Patient disposition and baseline characteristics

Thirty-seven patients were screened, of whom 36 were included and subsequently randomized (Figure 1). One patient dropped out of the study in the third week of treatment due to difficulties with NPH regimen adjustments. Three fMRI sessions had to be discarded (one because of excessive patient movement, two due to technical problems). Consequently, given the cross-over design, the corresponding sessions of these patients had to be excluded as well. Thus, 32 T1DM men were included in fMRI analyses (Table 1). Of these patients, 19 previously used insulin glargine, 10 ID and 3 NPH insulin before inclusion in the study.

**Table 1:** Patient characteristics.

N	32
Age (years)	36.3±9.4
Diabetes duration (years)	13.0±8.6
Body weight (kg)	83.3±13.9
BMI (kg/m2)	25.2±3.3
Systolic blood pressure (mmHg)	118±10.3
Diastolic blood pressure (mmHg)	77.8±7.1
HbA1c (%) (mmol/mol)	7.4±0.6 (57±6.6)
Total Cholesterol (mmol/L)	4.5±0.6
HDL Cholesterol (mmol/L)	1.5±0.4
LDL Cholesterol (mmol/L)	2.5±0.5
Triglycerides (mmol/L)	1.1±0.5
Urine albumin: creatinine ratio (mmol/mg)	1.1±2.7

### Clinical effects of ID versus NPH

ID decreased, whereas NPH increased body weight in T1DM patients (Table 2). Daily insulin doses and HbA1c were similar after each treatment period. Mean glucose and insulin levels during fMRI are listed in Table 2. In the subgroup of patients that consented to undergo an LP (*n* = 11 paired LP), serum insulin levels were slightly, yet significantly higher after treatment with ID versus NPH (*p* = .04), while CSF insulin levels for ID were substantially higher than for (*p* = .003; Table 2); CSF to serum insulin ratios were 0.075 versus 0.12 for NPH and ID respectively (*p* = .1) Blood glucose levels and CSF glucose levels were not different between treatments. CSF routine analyses did not show any abnormalities.

**Table 2:** Patient characteristics at baseline and at 12 week of intervention.

	NPH	ID	*P*
**T1DM patients, n = 32**			
Body weight (kg), t = 0 weeks	83.4±13.7	83.7±13.8	0.3
Body weight (kg), t = 12 weeks	83.9±14.2	83.0±13.7	0.007
Mean change in body weight (kg)	0.54±2.0	−0.76±1.7	0.02
HbA1c (%) (mmol/mol), t = 0 weeks	7.3±0.6 (56±6.6)	7.4±0.7 (57±7.7)	0.6
HbA1c (%) (mmol/mol), t = 12 weeks	7.4±0.6 (57±6.6)	7.4±0.6 (57±6.6)	0.8
Mean change inHbA1c (%) (mmol/mol)	0.038±0.39 (0.4±4.4)	0.0031±0.42 (0.0±4.6)	0.8
Daily insulin dose (basal) (IU/day), t = 12 weeks	27.8±12.9	27.7±11.2	0.9
Daily insulin dose (aspart) (IU/day), t = 12 weeks	32.1±12.7	31.7±12.2	0.7
Mean blood glucose at MRI (mmol/L)	10.4±4.0	8.8±3.6	0.05
Serum insulin level at MRI (pmol/L)	74.4 (47.1–121.4)	93.6 (61.1–119.9)	0.2
**Subgroup (that underwent lumbar puncture), n = 11**			
Mean blood glucose at MRI (mmol/L)	11.4±3.9	9.5±4.3	0.2
Mean CSF glucose at MRI (mmol/L)	5.2±2.2	5.8±1.4	0.5
Serum insulin level at MRI (pmol/L)	64.6 (44.2–77.3)	68.7 (57.0–106.9)	0.04
CSF insulin level at MRI (pmol/L)	4.8 (4.4–5.0)	8.3 (7.4–8.6)	0.003

As assessed by the DTSQ, perceived hyperglycemia and hypoglycemia did not differ significantly between treatments. Patient satisfaction was significantly greater when using ID versus NPH. No differences in eating behavior were found (DEBQ). ID treatment trended towards less hunger, appetite and desire to eat after the scan compared to NPH treatment (*p* = .10, *p* = .07 and *p* = .09, respectively). During fMRI, patients treated with ID classified 87.8±4.6% of all food and non-food items correctly (indoor versus outdoor classification), compared to 89.0±6.4% with NPH insulin treatment (NS). Response times were similar in both treatment groups for all picture categories.

### Main results: CNS effects of ID versus NPH

In both treatment groups, increased activation (higher BOLD signal) was observed in the ventral visual stream (occipital lobe) when viewing pictures (all categories) compared to baseline (i.e. pictures of an arrow), and in the food versus non-food contrast as well. Irrespective of treatment, T1DM patients showed an increased activation in the left ventral insula upon viewing food versus non-food items (Table 3, main effects of task).

**Table 3:** fMRI data.

	NPH insulin						Insulin detemir					
	L/R	coordinates			Z score	*p* value	L/R	coordinates			Z score	*p* value
		x	y	z				x	Y	z		
**Main effect of task: All versus baseline**												
Occipital cortex	L	−30	−46	−17	>8	<0.001	L	−30	−85	13	>8	<0.001
	R	39	−79	13	>8	<0.001	R	36	−82	10	>8	<0.001
	R	30	−61	−17	>8	<0.001	R	39	−82	−5	>8	<0.001
**Main effect of task: Food versus non-food**												
Occipital cortex	L	−15	−82	−14	7.15	<0.001	L	−12	−88	−11	6.10	<0.001
	L	−12	−91	−5	7.05	<0.001	L	−12	−94	−2	5.87	<0.001
	R	9	−79	−8	7.19	<0.001	R	9	−82	−8	6.47	<0.001
Ventral insula	L	−30	26	−8	3.43	NS	L	−33	29	−5	2.65	NS
							R	36	26	10	3.71	NS
**Group effects: Food versus non-food: NPH > detemir**												
Insula	R	36	−10	−11	3.33	0.02						
	L	−39	−10	1	2.90	0.05						

Coordinates of peak cluster activity from the normalized brain based on the Montreal Neurological Institute (MNI) system; ROI, region of interest.

**Figure 3:**
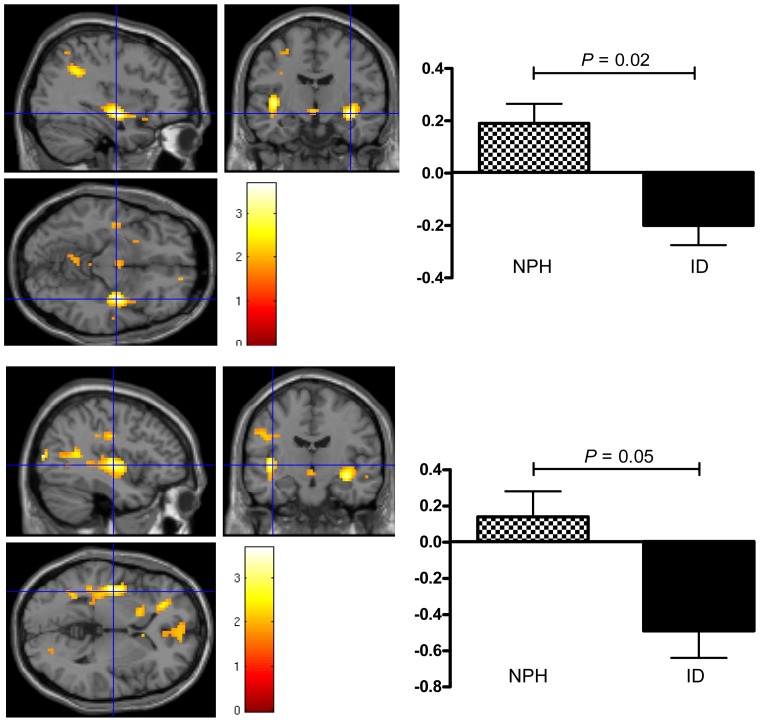
Increased activation in bilateral insula when watching food versus non-food pictures after treatment with NPH versus ID. SPM images for illustrative purposes. Increased activation after NPH treatment compared to ID treatment is shown in right (upper panel) and left insula (lower panel) respectively (crosshair); colour bar represents *t* value for paired Student's *t* test. In the graphs on the right the BOLD signal intensity (effect size) for each group is plotted (arbitrary units), mean ± SEM; NPH, NPH insulin; ID, insulin detemir.

Treatment with ID versus NPH resulted in significantly lower brain activation in bilateral insula when viewing food versus non-food items (*p* = 0.02, *Z* = 3.33 for right and *p* = 0.05, *Z* = 2.90 for left insula; Table 3, group effects; Figure 3), which was unaltered after correction for plasma glucose and insulin levels (data not shown). BOLD signal change in bilateral insula was positively associated with change in body weight after NPH (*r* = .51, *p* = .003 for right insula and *r* = .35, *p* = .05 for left insula). BOLD signal was not associated with change in body weight after treatment with ID (*r* = −.19, *p* = .30 for right insula and *r* = −.16, *p* = .40 for left insula). In the subgroup of patients that consented to undergo an LP, no correlations were observed between BOLD signal and CSF insulin levels or change in body weight after either treatment. There were no between-treatment differences when viewing high-calorie compared to low-calorie food items (data not shown).

## Discussion

Using a cross-over design in T1DM patients, we confirmed modest weight reduction after an ID-based versus NPH-based insulin treatment regimen [7], [24], but expanded these observations by showing significantly lower brain activation in bilateral insula in response to visual food stimuli during ID therapy. Interestingly, in a subgroup of patients, we found higher serum and CSF insulin levels after ID than after NPH treatment.

The insula, the region in which lower BOLD signal was observed, is relevant in the processing of food cues and craving for food [25] and is assumed to be involved in food choices [26]. Insulin acts in the CNS to induce satiety and inhibit food intake and weight gain, and insulin receptors are also present at high concentrations in the cerebral (insular) cortex, and are accessible via the CSF [27]. Therefore, ID may have both direct and indirect effects on the insula, ultimately resulting in reduced neural activation and suppression of the BOLD signal.

Whether the lower BOLD activation during visual food stimuli after ID treatment is cause or consequence of the observed weight loss cannot be determined from our study. Two prospective studies [15], [20] however, demonstrated that enhanced brain responses to food cues can predict future weight gain. Further evidence for a direct effect of ID on the CNS comes from studies using systemic infusions in mice and humans which resulted in acute changes in EEG and MEG as well as ensuing reductions in food intake [10]–[12].

Interestingly, a positive correlation was found between BOLD signal in bilateral insula and treatment-related body weight change only after treatment with NPH. Although no definite causal relationship may be inferred from this finding, it may be speculated that NPH modifies food-related activity in appetite regulating brain regions in a way that promotes weight gain.

Studies in mice investigating whether or not ID crosses the blood-brain-barrier used acute infusions and showed conflicting results: in one study a prefential brain tissue effect of ID was found after intravenous insulin injection, whereas in the other study it was shown that ID does not cross the mouse blood-brain-barrier at all [10], [28]. In humans, information on CSF insulin levels during treatment with various insulin formulations is lacking. Since (long-standing) T1DM is associated with absence of endogenous insulin production, patients with T1DM seem an attractive model to determine CSF insulin levels that should entirely reflect the result of exogenous administration of insulin. Although our study is the first to perform LP procedures to this end in T1DM, due to the associated discomfort and potential side-effects, a relatively small number of patients consented to undergo this procedure twice during the course of the study. Nevertheless, in this relatively small sample, increased CSF insulin levels were found in patients treated with ID versus NPH, in spite of comparable daily basal insulin doses, supporting the hypothesis that ID crosses the BBB more readily than NPH, or that clearance of ID from the CSF occurs at a slower rate.

In the present study we focused on ID action in the brain, but other mechanisms underlying its weight reducing effect have been proposed, e.g. increased energy expenditure and reduced frequency of hypoglycemia. However, in a randomized cross-over study [29] comparing ID and NPH insulin treatment no difference in energy expenditure between both treatments was observed. Since ID treatment results in more stable insulin levels and a greater effect on glucose metabolism in the liver than in peripheral tissues [30], it has also been suggested that the weight difference between NPH and ID is due to a reduced frequency of hypoglycemia with ID [31]. As patients not systematically reported all hypoglycemias (and snacking) during the present study, we cannot rule out that a reduced hypoglycemia (and snacking) frequency caused the differences in body weight. However, this hypothesis is unlikely to fully explain the weight-sparing effect of ID, as supported by the fact that other studies demonstrated that insulin glargine, when causing the same frequency of hypoglycemia as ID, resulted in more weight gain.

Limitations of the study include the non-blinded study design: NPH and ID can be visually distinguished as NPH is a cloudy solution that needs to be mixed before injection, while the ID preparation is available as a clear solution. However, worldwide, NPH is the standard (intermediate) long-acting human insulin and therefore the best active comparator. Moreover, since subjects were not aware of the food-related nature of the experiment, it is unlikely that knowledge of the type of insulin treatment has influenced the BOLD effects in our study. Additionally, possible confounders that could have accounted for the observed differences in CNS responses to food cues include HbA1c or prevailing glucose and/or insulin plasma levels. We deliberately chose to study patients in a real-life setting as compared to an acute exposure (e.g. clamp condition), since it is unclear whether this artificial condition can be extrapolated to real-life conditions in which weight differences are observed. In the present study, adjustment for glycemic variables and insulin levels did not alter the fMRI-measured results. Preferably, a cross-over trial should have a wash-out period to avoid carry-over effects. However, since all type 1 diabetes patients need some type of long-acting insulin every day, insulin washout was not an option. An alternative option to minimize carry-over effects is to lengthen treatment periods [32]. In our study, we lengthened the period of both treatments from the originally planned 8 weeks to 12 weeks. Since the duration of action of NPH and ID on glucose metabolism is less than 24 h, we expect that lengthening the treatment period to 12 weeks was sufficient. Weight gain associated with insulin treatment is relevant for both T1DM and type 2 diabetic (T2DM) patients although this side effect of insulin treatment may be more relevant in T2DM patients who generally are already overweight or obese. Although it is tempting to generalize our findings to T2DM, this study should be repeated in T2DM patients in future investigations.

In summary, the present study expands the limited data available, describing the effects of insulin on the human brain. We showed that a 12-week treatment with ID, compared to NPH, resulted in weight loss in T1DM patients, which was paralleled by decreased brain activation in the bilateral insula in response to visual food stimuli. Furthermore, ID versus NPH treatment resulted in elevated CSF insulin levels. These findings support the hypothesis that the weight sparing effect of ID may, at least in part, be mediated by its enhanced action in the CNS that interferes with food-related activation in appetite regulating brain regions.

## Supporting Information

Checklist S1 **CONSORT checklist.**
(DOC)

Protocol S1 **Detailed study protocol.**
(PDF)
