# Supplementary material for: Effects of Insulin Detemir and NPH Insulin on Body Weight and Appetite-Regulating Brain Regions in Human Type 1 Diabetes: A Randomized Controlled Trial
Source: PLoS One. 2014 Apr 16;9(4):e94483. doi: 10.1371/journal.pone.0094483 (PMC3989203; doi:10.1371/journal.pone.0094483)
Supplement: Protocol S1 — Detailed study protocol. (PDF) [file pone.0094483.s002.pdf]

Confidential

Effects of INsulin dEtemiR and neutral protaminE hagedorn (nph) insulin on BRain glucOse metabolism: a study in persons with type 1 diabetes INcEREBRO

Department of Endocrinology/Diabetes Center, VU University Medical Center, PO Box 7057, 1007 MB, Boelelaan 1117, Amsterdam, The Netherlands. Telephone: +31 20 4440533, Telefax: +31 20 4440502

Investigators:

Endocrinology	M.Diamant (PI)
	M.L. Drent
	L.W. van Golen
Nuclear Medicine and	A.A. Lammertsma
PET Research	M.C. Huisman
Psychiatry	D.J. Veltman
Radiology	F. Barkhof
	E. Sanz-Arigita
Neurology	Ph. Scheltens
Psychology	J.B. Deijen

Sponsor's protocol code number: DC2007Det001

EudraCT number: 2007-007255-13

CWO-ICEN number: 07-48

1.1. GENERAL STUDY INFORMATION

**SPONSOR**	M. Diamant<br>Department of Endocrinology/Diabetes Center<br>De Boelelaan 1117<br>PO Box 7057<br>1007 MB Amsterdam<br>Telephone: +31 20 4440533<br>Telefax: +31 20 4440502

**RESEARCH INSTITUTE**	VU University Medical Center<br>Department of Endocrinology/Diabetes Center<br>De Boelelaan 1117<br>PO Box 7057<br>1007 MB Amsterdam<br>Telephone: +31 20 4440533<br>Telefax: +31 20 4440502

**PRINCIPAL INVESTIGATOR**	M. Diamant<br>Department of Endocrinology/Diabetes Center<br>De Boelelaan 1117<br>PO Box 7057<br>1007 MB Amsterdam<br>Telephone: +31 20 4440533<br>E-mail: m.diamant@vumc.nl

1.2. TABLE OF CONTENTS

1.1 General study information……………………………………………p. 2
1.2 Table of contents……………………………………………………p. 3
1.3 Protocol summary…………………………….………………..p.5
2. Background…………………………………………………………..p. 6
3. Study hypothesis and study endpoints…………………………………….p. 10
3.1 Study hypothesis……………………………………………..p. 10
3.2 Study endpoints……………………………………………p. 10
4. Methods……………………………………………………………p. 12
4.1 Study design ……………………………………………p. 12
4.2 Study population……………………………………………..p. 12
4.3 Exclusion criteria……………………………………………..p. 12
4.4 Recruitment and screening examination……………………..p. 13
4.5 Study outline………………………………………………p. 14
5. Measurements……………………………………………………...p. 15
5.1 Blood, urine and CSF sampling……………………………p. 15
5.2 Protocol PET…………………………………………….p. 18
5.3 Protocol structural and functional MRI...p. 21
5.4 Psychological assessment………………………………………..p. 23
6. Statistical considerations……………………………………………...p. 24
6.1 General considerations……………………………………….p. 24
6.2 Analysis population…………………………………………p. 24
6.3 Primary study endpoints………………………………………….p. 24
6.4 Justification of sample size………………………………………p. 24
6.5 Data analysis…………………………………………………...p. 25
7. Study medication…………………………………………………p. 25
7.1 Storage and provision………………………………………….p. 25
7.2 Randomisation of study medication…………………………..p. 25
7.3 Dose titration……………………………………………….p. 26
8. Study drug discontinuation/ withdrawal from the study……………….…...p. 26
9. Study completion……………………………………………..……p. 27
10. Laboratory procedures/ total blood volume……………………….....p. 27
11. Informed consent form for trial subjects……………………….…….p.27
12. Financial compensation……………………………………………....p. 28
13. Ethics………………………………………………………………p. 28
14. Publication policy…………………………………………………….p. 28
14. Insurance……………………………………………………………..p. 29
15. Abbreviations……………………………………………………...p. 30
16. References……………………………………………….…….p. 31

Appendix 1: Flow chart…………………………………………………….……………..…...p. 38

Appendix 2: Blood, CSF, urine…………………………………………….…………….p. 39

Appendix 3: Safety considerations and reporting……………………………………………..p. 40

Introduction…………………………………………………..…………………p. 40

Background…………………………………………………………..…………….p. 40

Definitions of - and the procedures for reporting - an Adverse Event (AE)…………p. 41

Definitions of - and the procedures for reporting - a Serious Adverse Event (SAE)... p.41

Reporting of SUSARS…………………………………………………………………..p. 42

Yearly report…………………………………………………………………………….p. 42

Contact details…………………………………………………………………..p. 43

Appendix 4: Healthy controls…………………………………………………………………….p. 44

1.3 PROTOCOL SUMMARY

Background

Intensive insulin therapy improves the long-term outcome of diabetes patients, but is also associated with weight gain, a very unwanted side-effect. Insulin detemir is a relatively new basal insulin analogue, which consistently has been shown to result in less weight gain as compared to insulin NPH. This observation has so far not been explained. However, a possible mechanism could be an enhanced effect of insulin detemir *in cerebro* in communicating satiety signals. Since insulin receptors are abundantly present in the brain, it is of interest to assess whether reduced weight gain is associated with increased concentrations of insulin detemir in the cerebrospinal fluid (CSF) and whether there are differences in glucose metabolism and blood flow in brain areas potentially involved in appetite regulation. Positron emission tomography (PET) scanning allows the measurement of cerebral glucose metabolism and blood flow and functional magnetic resonance imaging (fMRI) enables the assessment of activation of brain regions involved in appetite regulation.

Objectives

The aim of this study is to test the hypothesis that subcutaneous administration of insulin detemir, as compared to NPH insulin, leads to a more pronounced effect on brain glucose metabolism and blood flow in brain regions associated with appetite regulation. Furthermore, we would like to explore whether insulin detemir, as compared with NPH insulin, leads to higher insulin concentrations in the CSF, whether it affects appetite control in specific brain areas and whether the changes in appetite control are related to changes in brain glucose metabolism, blood flow and/or insulin concentration in the CSF.

Study design

Randomised, open-label cross-over study. Type 1 diabetic patients will be treated on a basal-bolus regimen for two periods of 12 weeks, starting with insulin detemir or NPH insulin QD, administered in the evening, the prandial insulin being insulin aspart. PET and fMRI measurements and a lumbar puncture will be performed in the last week of both treatment periods.

Study population

Forty right-handed male type 1 diabetic patients with a disease duration of >1 years, aged 18-60 years, in good to moderate glycaemic control (HbA1c ~7,5%) will be enrolled.

Parameters

Cerebral glucose metabolism and cerebral blood flow will be measured with $[^{18}F]$fluorodeoxyglucose (FDG) PET and $H2^{15}O$ PET respectively. Insulin detemir and NPH insulin concentrations in the CSF will be measured. Assessment of brain activity in appetite-related regions will be determined by fMRI.

2. BACKGROUND

Intensive insulin therapy, targeting normoglycaemia in patients with diabetes, improves long-term outcome, but increases the risk of hypoglycaemia [1, 2] and is associated with body weight gain both in type 1 [3-6] and type 2 diabetes [7, 8].

Insulin detemir (Lys-B29 (N-tetradecanoyl) des (B30) human insulin) was the first clinically available acetylated insulin analogue; the amino acid threonine at B30 is removed and a 14-carbon, myristoyl fatty acid is acylated to lysine at B29 [9]. The protracted action of detemir is primarily achieved through slow absorption into blood. Diheximerization and albumin binding of hexameric and dimeric detemir prolongs residence time at the injection depot. Further retention of detemir occurs in the circulation where albumin binding causes buffering of insulin concentration.
Detemir has consistently been shown to have several beneficial properties relative to NPH insulin: insulin detemir results in less pharmacodynamic and pharmacokinetic variability [10-13] [14]and less frequently occurring nocturnal hypoglycaemia [15-19]. Furthermore, insulin detemir has repeatedly shown to result in less weight gain than NPH insulin, in spite of equal improvement of glycaemic control in type 1 and type 2 diabetes [15, 16, 19-23]. This observation has so far not been explained. However, several possible mechanisms are described, e.g. a preferential effect on liver metabolism of insulin detemir, insulin detemir causing reduced defensive snacking because of less hypoglycaemia and an enhanced effect of insulin detemir *in cerebro* in communicating satiety signals.

Detemir & weight stability: the liver? A potential mechanism, explaining body weight stability, could be a preferential effect on liver metabolism of insulin detemir. In comparison with other insulins, detemir has been shown to have a relatively greater effect on the liver than on peripheral muscle and adipose tissue. Because no significant barrier exists between blood plasma in the sinusoid and the hepatocyte plasma membrane, insulin detemir, both free and albumin-bound, is taken up continuously by the hepatocytes. In contrast, the binding to albumin retards the transfer of insulin detemir from the circulation into the adipose tissue and skeletal muscle. Therefore, the albumin binding of detemir increases the hepatic-peripheral insulin gradient, mimicking the physiological, non-diabetic state. Thus, in order for detemir to exert similar blood glucose control, its effect on and extraction by the liver may in relative terms be higher, resulting in reduced endogenous glucose production, whereas its peripheral action, including anti-lipolytic activity, is lower [24] [12, 25].

Detemir & weight stability: less caloric consumption? An alternative hypothesis explaining the decreased weight gain is detemir causing less hypoglycaemia and therefore less defensive snacking [15-17, 20, 26, 27]. This hypothesis is unlikely to fully explain the weight-sparing effect, as supported

by the fact that glargine, when causing the same frequency of hypoglycaemia as detemir, resulted in more weight gain [28, 29].

Detemir & weight stability: the brain? The relative weight stability of insulin detemir therapy can also be attributed to altered kinetics of insulin signalling *in cerebro*. Since insulin receptors are abundantly present in the brain [30, 31], it is of interest to assess whether reduced weight gain is associated with increased concentrations of insulin detemir in the cerebrospinal fluid (CSF) and whether there are differences in glucose metabolism in brain areas potentially involved in appetite regulation.

Recently, it has been shown by Hennige et al. [32], by comparing intravenous administration of insulin detemir and human insulin in mice, that insulin action in hypothalamic and cerebrocortical centres was enhanced when compared to peripheral action, due to a higher insulin detemir concentration in the brain. Moreover, epidural EEG in these mice displayed increased cortical activity in the ones on insulin detemir. These results suggest the existence of a tissue-specific (brain) kinetics of insulin detemir, at least in mice. According to the authors, the enhanced insulin detemir action *in cerebro* could possibly be explained by an accelerated insulin uptake due to the attachment of the fatty acid chain to position B29 on the insulin detemir molecule, which may result in a different penetration of the blood brain barrier and in different binding properties in the central nervous system.

The observation of enhanced insulin signalling in the brain using insulin detemir is of particular interest in the context of former studies in humans using intranasal insulin, which enters the cerebrospinal fluid compartment without affecting circulating insulin levels in the blood stream [33, 34]. In healthy, normal-weight men, the administration of intranasal insulin resulted in a loss of body weight and a reduction of body fat after an 8-week treatment; the women gained weight [35]. In obese men, intranasal insulin did not result in weight loss; it did however improve declarative memory and mood [36]. The authors conclude that obesity in men is associated with central nervous resistance to the adiposity signal insulin and that this defect is likely to contribute to the persistence of obesity in spite of elevated levels of circulating insulin in obese patients. A recent study by Tschritter et al. [37] reveals that insulin modulates cerebrocortical activity in lean humans, whereas in obese individuals these effects were not detectable. In this study, cerebrocortical insulin action was positively correlated with peripheral insulin sensitivity and inversely correlated with measures of obesity. Furthermore, a genetically determined and obesity-related cerebrocortical insulin resistance could be demonstrated. A study by Anthony et al. [38], using positron emission tomography (PET), also shows insulin resistance in brain glucose metabolism, especially in regions subserving appetite and reward, in humans with peripheral insulin resistance.

These data provide strong evidence for a negative feedback signal of insulin (at least in lean men) in the regulation of body weight. As in these studies the CSF insulin concentrations were not measured, it could also be that the difference in brain glucose metabolism was caused by a different insulin concentration *in cerebro*. A decreased CSF: plasma insulin ratio with increasing body weight was found by Kern et al. [39], indicating that obesity in humans, as in animals is characterised by a relative central nervous insulin deficit.

The central processing of food related stimuli has been studied by Morris et al. in healthy volunteers using PET [40]. In the fasting state, the recognition of food related (compared to non-food related) stimuli was better than in the state of satiety, especially in the amygdala and the orbitofrontal cortex. Our research group studied this process in lean healthy volunteers using functional MRI and we were able to confirm these results (data submitted for publication). Tataranni et al. [41] and Small et al. [42] also studied this process in healthy persons, by using H2O-PET.

All together, it has been clearly demonstrated that insulin affects brain function [43], feeding behaviour [44-54], and also auditory evoked potentials [55]. Whether these effects are mediated (partly) via an effect on brain glucose metabolism (insulin-stimulated glucose metabolism) is unclear. Studies applying PET in healthy [56, 57] and diabetic [58] subjects did not find an effect of increasing insulin levels on regional cerebral glucose uptake. Also the results of a magnetic resonance spectroscopy study revealed that insulin has no acute effect on glucose transport or metabolism in humans [59]. Based on the lack of effect of hyperinsulinaemia, it has been concluded from these studies that human brain glucose metabolism is not insulin sensitive. However, work from Bingham et al. [60] has suggested that basal insulin levels may influence brain glucose uptake in a region-specific manner, with the greatest effect in cortical regions and the least effect in the cerebellum. Considering the published data showing no effect of increasing circulating insulin levels above fasting levels, it could be concluded that brain glucose metabolism is maximally stimulated at fasting insulin concentrations. Nevertheless, these results are in contrast with the evidence that insulin has an effect on brain function and appetite control. These observations have clinical implications in view of suggestions that different insulin preparations may differentially affect various brain functions.

Other potentially important determinants of insulin action in the brain could be considered, such as microvascular disease, oxidative stress, inflammation, and vascular insulin resistance [61]. Micro- and macrovasculopathy in diabetes – and presumably also the onset and progression of Alzheimer's disease [62, 63] - are promoted by the formation of advanced glycation end-products (AGE's) and advanced lipoxidation end-products (ALE's) [64-70]. Because glucose levels in the central nervous system (CNS) are lower than in the peripheral blood, the level of AGE's in the cerebral spinal fluid is likely to be different from plasma levels, as shown by Ahmed et al. [63]. Also, the turnover of proteins

in plasma and CNS differ. Therefore, it would be interesting to determine AGE's in plasma as well as in CSF. Other variables that are possible confounders or modifiers of the natural course of cognitive decline in DM1, such as Aß42 and Tau (associated with (mild) cognitive impairment, particularly in Alzheimer's disease), p-tau and neurofilaments [71]could also be interesting to determine. There is evidence that insulin modulates the level of Aß42 [72] and Tau [73]. Also, insulin has a positive effect on cognitive function, as shown by studies of intranasal insulin in Alzheimer patients [74], as well as in healthy volunteers [43, 75] and obese men [36]. Another variable that is associated with cognitive function [76-78] as well as lipid profile and coronary risk [79] is the APOE phenotype. Determination of MBL (mannose-binding lectin) status could be useful to identify patients at increased risk of developing micro- and macrovascular complications.

To summarize, intensive insulin therapy improves long-term outcome in diabetes patients, but is also accompanied by an increase in bodyweight. Compared to other NPH insulin, insulin detemir has been shown to cause less weight gain, an observation which still remains to be explained. As obesity is becoming a major health problem, it would be very interesting to elucidate the physiology of how detemir prevents diabetic patients from gaining weight. A possible explanation could be an altered kinetics of insulin signalling or processing *in cerebro*, which is the focus of this research project. By determining cerebral glucose metabolism and blood flow, insulin concentration in CSF and changes in activity in specific brain areas involved in energy metabolism and feeding behaviour, we hope to find an explanation for this interesting and promising observation.

3. STUDY HYPOTHESIS AND STUDY ENDPOINTS

3.1 Study hypothesis:

Subcutaneous treatment with insulin detemir, as compared to NPH insulin, leads to similar glycaemic control but less weight gain in patients with T1DM due to improved insulin action in brain regions involved in energy metabolism and feeding behaviour. To test this hypothesis we will address the following research questions:

1) Does a 12-wk intervention with insulin detemir relative to 12-wk NPH insulin therapy in T1DM patients lead to:
    a) An increased glucose metabolism and blood flow in brain regions associated with appetite regulation (e.g. amygdala, orbitofrontal cortex, insula, hypothalamus)?
    b) Increased insulin concentrations in the CSF?
    c) Changes in control of appetite in specific brain areas (e.g. amygdala, orbitofrontal cortex, insula, hypothalamus)?
    d) Less weight gain?
2) Are the observed effects on appetite control associated with changes in brain glucose metabolism, blood flow, insulin concentration in the CSF and/ or weight change?

3) Are the observed effects of insulin on appetite control, glucose metabolism and blood flow, concentration of insulin in CSF and change in body weight, dependent on duration of diabetes and/or other patient related factors e.g. the presence of (subclinical) microvascular disease?

4) Are there differences in the effects of insulin detemir and NPH on various dimensions of quality of life, e.g. mood and eating behaviour?

3.2 Study endpoints

Primary study endpoints:

I. Cerebral metabolic rate of glucose (CMR_{glu}) expressed in umol/100mg/min, in brain regions associated with appetite regulation (e.g. amygdala, orbitofrontal cortex, insula, hypothalamus), as determined by FDG-PET scanning

II. Cerebral blood flow (CBF), expressed in ml/100mg/min, in brain regions associated with appetite regulation (e.g. amygdala, orbitofrontal cortex, insula, hypothalamus), as determined by H2O-PET scanning

Secondary study endpoints:

I. Insulin concentrations in the CSF

II. Brain activity in specific regions, involved in control of appetite (e.g. amygdala, orbitofrontal cortex, insula, hypothalamus), as determined by fMRI

III. Weight change

Other parameters to be determined:

a. The association of the observed effects on appetite control, with changes in brain glucose metabolism, blood flow, insulin concentration in the CSF and weight change

b. The relationship of the observed effects of insulin on appetite control, glucose metabolism and blood flow, insulin concentration in CSF and body weight, with duration of diabetes and/or other patient related factors, e.g. the presence of (subclinical) microvascular disease

c. Possible differences in the effects of insulin detemir and NPH on various dimensions of quality of life, e.g. mood and eating behaviour

Exploratory variables: free fatty acids (FFA’s), ketone bodies, lactate, markers of microvascular disease and vascular insulin resistance (AGE’s: CML, CEL, 3DG), oxidative stress (malondialdehyde, ox-LDL, F2-isoprostanes), inflammation (IL6, TNFalpha, CRPhs); appetite/food-intake related hormones (ghrelin, leptin); markers of cognitive decline: Aß42, tau, p-tau; markers of increased risk of cognitive decline (APOE-genotype) and micro-/macrovascular complications (mannose-binding lectine, MBL).

4. METHODS

4.1. Study design

A randomised, open-label cross-over study will be performed. This will allow to compare the results of two treatment regimens on the same group of patients, accounting for possible inter-individual differences in pharmacodynamics and –kinetics of the insulins used, by each individual being its own control. After a 4-week run-in period, during which patients continue their usual insulin, they will be treated for 12 weeks with either insulin detemir or NPH QD, administered in the evening, in combination with the prandial insulin analogue insulin aspart TID. A lumbar puncture, PET and fMRI measurements will be performed in the last week of this period, after which cross-over will take place and another 12 weeks of treatment, followed by the same procedures.

4.2. Study population

Forty right-handed male type 1 diabetes patients with a disease duration >1 year, aged 18-60 years, in good to moderate glycaemic control (HbA1c ~7.5%) will be enrolled. This patient selection (diabetes type 1) avoids inclusion of persons with endogenous insulin secretion. Because body mass index (BMI) has a possible modifying effect on cerebral glucose metabolism and insulin signaling [35-39, 74, 80-86], participants with a wide BMI-range (18-35 kg/m2) will be included. As appetite and feeding behaviour are influenced differently in women compared to men [35, 87] women will not be included in this study.

4.3. Exclusion criteria

- Recent onset DM1 (disease duration < 1 years)
- BMI < 18 and > 35 kg/m2
- Type 2 diabetes
- History of major heart or renal disease
- Severe untreated proliferative retinopathy
- History of recurrent severe hypoglycaemia (> 3 episodes in the last year, requiring i.m. glucagon or i.v. glucose administration)
- (History of) brain disorders
- Alcohol abuse (defined as more than four units alcohol on average per day)
- (History of) use of MDMA, cocaine, heroin, methylphenidate, current regular use of cannabis, or history of regular use on a daily basis for at least 5 years, current use of benzodiazepines, non-selective beta-blockers, oral steroids (>7.5 mg/day), oral anti-coagulants
- Current psychiatric disease/treatment
- (History of) eating disorders

- History of severe head trauma accompanied by loss of consciousness
- Any endocrine disease not well-controlled for at least three months
- Inability to undergo MRI (such as claustrophobia, metal implants, BMI>35)
- Visual acuity < 0.3 at the last ophthalmologic examination
- Known or suspected allergy to trial product or related products

4.4. Recruitment and screening examination

Subjects will be recruited primarily from our outpatient clinic. During a screening visit, subjects will be informed extensively about the study. After having obtained written informed consent, a physical examination and blood sampling will be done. Autonomic neuropathy will be assessed using cardiovascular Ewing's tests [88]. At every visit, body weight will be measured.

The severity of diabetic sensorimotor polyneuropathy (DSP) will be quantified by The Toronto Clinical Scoring System [89]. This is a valid instrument to reflect the presence and severity of DSP and correlates well with other, more invasive, measures such as sural nerve morphology and electrophysiology.

Patients will be screened for retinopathy, which will be quantified by an ophthalmologic examination of each eye. Two pictures (Topcon camera) of the retina of each eye will be made, following the application of mydriatric eye drops. The criteria for diabetic retinopathy (DRP) classification (EURODIAB classification [90]) will be the condition of the most severely affected eye.

Patients will be asked to fill out a questionnaire regarding their current eating behaviour, to exclude eating disorders and a questionnaire about their current state of mood. Patients will be interviewed using a questionnaire to assess the number, frequency and severity of hypoglycaemic episodes during treatment with insulin, as described before [91, 92]. Severe hypoglycaemia will be defined, according to the criteria used in the Diabetes Control and Complications Trial, as an episode that requires external assistance to aid recovery [93]. Hypoglycaemia history will be graded on a four- point scale: 1 = no previous episodes of severe hypoglycaemia, 2 = one or two previous episodes, 3 = three to five previous episodes; 4 = more than five previous episodes. Patient’s history of hypoglycaemia will be verified if possible, by scrutinizing notes made by the patient’s general practitioner or hospital case records (when available). In order to record episodes of hypoglycaemia during the time period of the study, patients will be asked to register each hypoglycaemic episode, and rapport these at each visit, with severe hypoglycaemic episodes reported as soon as possible.

4.5. Study outline

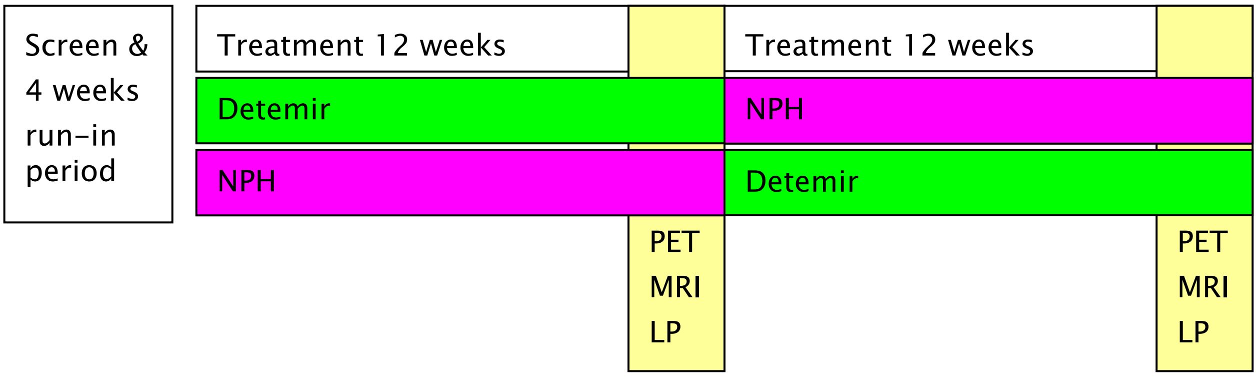

The general study outline is presented in the figure above (a more detailed scheme is presented in Appendix 1). At visit 1, a voluntary, signed and dated informed consent will be obtained from the subjects prior to any trial related procedure. Participants will be screened for eligibility and enrolled into a run-in period of four weeks, during which they continue their usual insulin therapy. Further tests (Ewing test, assessment of DSP, fundoscopy) will be done, blood will be drawn (if this was not done at our hospital during the previous three months), insulin therapy will be reviewed, a detailed history will be taken and a physical examination will be done. At visit 2 blood will be drawn to measure HbA1c. Patients will be randomized to initial treatment with insulin detemir or NPH; this insulin is to be used once daily for the first 12 weeks, the prandial insulin will be the same during all study period for all participants (insulin aspart). Telephone contacts will be made during both treatment periods, to take a recent history, monitor adverse events and adjust insulin dose if necessary, which is of specific importance when the PET scan, MRI and LP will take place, at euglycaemic conditions.

In the last week of the first treatment period (visit 3), measurements will be performed at our institution to assess cerebral blood flow and glucose metabolism, by PET-scanning. In the same week, additional fMRI studies and a lumbar puncture will be performed (visit 4). At both visits, patients will have to fill out a short questionnaire concerning mood and feeling of satiety. Afterwards, patients will receive the new (the other) insulin, which is also to be used for 12 weeks, after which all procedures will be repeated (visit 5 and 6). At the end of the study period, patients can resume their previously used insulin; this will be discussed during the last visit.

5. MEASUREMENTS

5.1. Blood, urine and CSF sampling (see Appendix 2)

Blood will be drawn for the following measurements:

Screening/ safety: HbA1c, haemoglobin, leucocytes, thrombocytes, creatinine, liver enzymes (AF, γ-GT, ALT), TSH, lipid profile

Study parameters: glucose, insulin (specific assays), albumin, leptin, ghrelin

Exploratory variables: advanced glycation endproducts (AGE's, in particular N^{ε}-(carboxymethyl)-lysine (CML), N^{ε}-(carboxyethyl)-lysine (CEL) and 3-deoxyglucosone (3DG)), markers of oxidative stress (Malondialdehyde, Ox-LDL) and inflammation (IL6/TNFα/CRPhs), ketone bodies, lactate, APOE genotype and MBL (mannose-binding lectin)

Cerebrospinal fluid (CSF) will be obtained to measure:

Safety/ standardisation: total cells, total protein

Study parameters: glucose, insulin (specific parameters), albumin

Exploratory variables: Aß42, tau, p-tau, and AGE's (CEL, CML, 3DG)

Urine: a 24-hr urine collection will be obtained:

Screening/ safety: microalbumin, creatinin

Exploratory variable: F2-isoprostanes (oxidative stress)

INcEREBRO										
**Flow Chart**	**Visits**									
	**1**	**2**	**TC1**	**3**	**4**		**TC2**	**5**	**6**	**E**
	**Screening**	**Randomi sation**	**Tel call**	**Measurements**		**Cross-over**	**Tel call**	**Measurements**		**Evalua tion**
	Week -4	Week 0	Week 2	Week 12	Week 12		Week 14	Week 24	Week 24	Week 26
		Detemir				NPH				
		NPH				Detemir				
Informed Consent	X									
Incl./Excl. Criteria/ demography	X									
Start of study insulin		X				X				
Insulin Therapy (e.g. titration, instruction, monitoring hypoglycaemic events)	X	X	X	X	X		X	X	X	X
Patient History<br>– past<br>– recent	X 	 <br>X	 <br>X	 <br>X	 <br>X		 <br>X	 <br>X	 <br>X	
Physical Examination<br>– General<br>– DSP<br>– Ewing test<br>– Fundoscopy	X<br>X<br>X<br>X			X				X		
Vital Signs (incl. body wt)	X	X		X	X			X	X	X
Monitoring (S) AE's			X	X	X		X	X	X	
Concomitant medication	X	X	X	X	X		X	X	X	X
PET-scan				X				X		
fMRI-scan					X				X	
Blood measurements<br>– screening<br>– safety; study parameters	X 	 <br>X		 <br>X	 <br>X			 <br>X	 <br>X	
Lumbar puncture					X				X	
Questionnaires:<br>EDI-II; CES-D<br>POMS, Likert<br>DEBQ	X 	 <br>X		X<br>X	X 			X<br>X	X 	
Urine analysis		X		X				X		

At visit 3 and 4, both in the last week of the assigned treatment, participants will visit our institution in the early morning after an overnight fast from 10 PM. In the evening preceding these visits, patients will inject their dose of insulin detemir or NPH subcutaneously, just as they have done the preceding weeks. Telephone calls will be made to instruct patients what insulin dose to use in order to optimize their blood glucose. Approximately nine hours following the evening injection of the basal insulin (detemir or NPH), PET scanning/fMRI scanning will be performed. Morning (prandial) insulin injection will be administered after fMRI and PET scanning. Blood and CSF samples (lumbar puncture) will be taken (at visit 4 and 6) after MRI scanning.

CSF will be obtained by a lumbar puncture. Patients will be placed in a lateral decubitus position. A 25G atraumatic spinal needle will be inserted, and approximately 10 ml of CSF will be collected. Because this very thin needle will be used, the risk of post-punctional headache is very small [72, 94]; however, patients will be informed about this risk.

5.2. Protocol PET

After 12 weeks of treatment, patients will visit the Radionuclide Centre (RNC) in the early morning; they will be instructed to be in a fasting state and not to inject their prandial insulin.
Blood glucose values should be maintained at values ranging from 5-10 mmol/l. Therefore, blood glucose values will be checked (and adjusted, if necessary, by the infusion of 5% glucose) before and after testing using a finger prick.

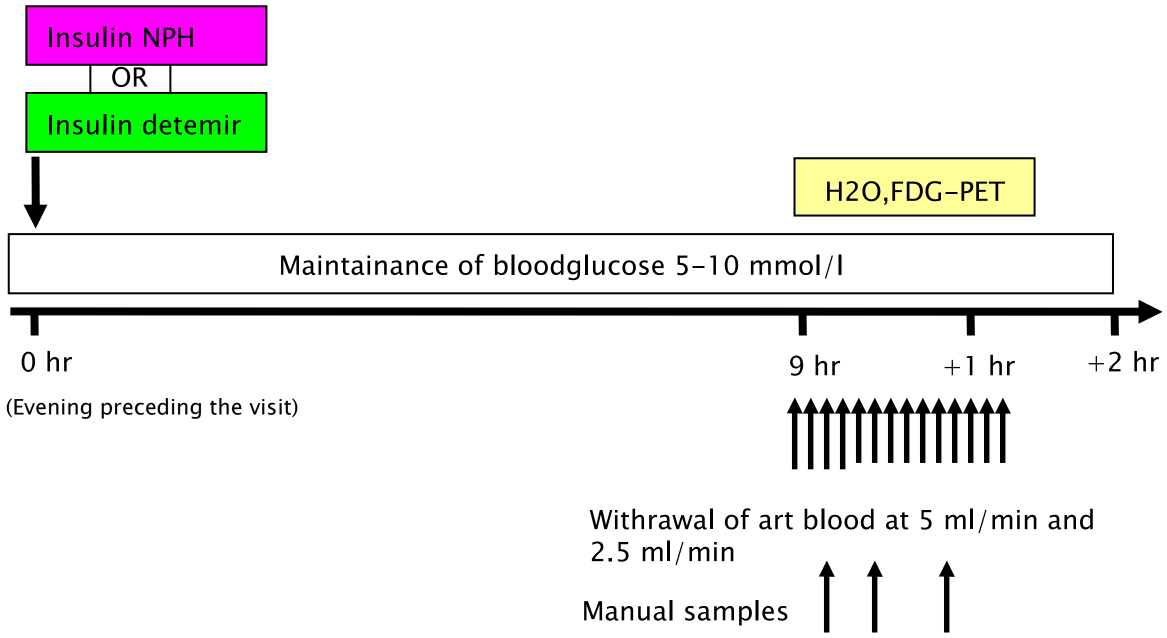

All scans will be acquired at the same time of the day to minimise diurnal variations. All patients will receive an indwelling radial artery cannula. This arterial cannula will be inserted under local anaesthetics by an experienced clinician and will be used for blood sampling. In addition, a venous cannula will be inserted, which will be used for tracer injection (and glucose 5% if necessary). Subjects will be transferred to the scanning room and positioned on the scanner bed. They will be made comfortable in a supine position on the scanner table. The head will be fixated to reduce movement artefacts and, using laser beams, positioned within the centre of axial and transaxial field of views, such that the orbito-meatal line is parallel to the detectors.

Scanning protocol

PET scans will be performed on an ECAT HRRT (High Resolution Research Tomograph) scanner (Siemens/CTI), a dedicated whole-brain and small animal PET scanner, with design features that enable high image spatial resolution combined with high sensitivity; the scanner field of view (FOV) is 312 mm in diameter and 250 mm in axial length. The HRRT is the first commercially available

scanner that utilizes a double layer of LSO/LYSO crystals to achieve photon detection with depth-of-interaction information [95].

First a 10 minutes transmission scan (TS) will be performed in 2D mode using a single photon emitter moving point source. These data will be used to correct the subsequent emission scans for photon attenuation.

Following the transmission scan, 1100 MBq of $[^{15}O]H_2O$ will be injected intravenously, simultaneously starting a dynamic emission scan (ES) in 3D mode. This dynamic scan will have a total duration of 10 minutes and will allow to determine cerebral blood flow (CBF), expressed as ml/100mg/min, in specific brain regions associated with appetite regulation. Throughout the scan, arterial blood will be withdrawn continuously at a rate of 5 ml/min, using an on line detection system (Veenstra Instruments, Joure, Netherlands), cross-calibrated against the PET scanner.

Next, 185 MBq of $[^{18}F]$FDG will be injected intravenously, again simultaneously starting a dynamic emission scan in 3D mode with a total duration of 60 minutes. This allows to determine cerebral metabolic rate of glucose (CMRglu), expressed as umol/100mg/min, in specific brain regions. Arterial blood will be withdrawn continuously at a rate of 5 ml/min for the first 10 minutes and 2.5 ml/min thereafter. At set times, continuous withdrawal will be interrupted briefly for the collection of blood samples, which will be used to calibrate the on-line detection system and to estimate plasma-to-whole-blood ratios of radioactivity. After each sample, the line will be flushed with heparinised saline. A maximum of 250 ml blood will be withdrawn during an entire PET session. Total scanning time: 80 minutes.

Treatment 12 w		Treatment 12 w	
Detemir		NPH	
NPH		Detemir	
	<u>PET</u><br>2D TS (10')<br>1100 MBq H2O<br>3D dyn ES (10')<br>185 MBq FDG<br>3D dyn ES (60')<br><br><u>Total: 80'</u>		<u>PET</u><br>2D TS (10')<br>1100 MBq H2O<br>3D dyn ES (10')<br>185 MBq FDG<br>3D dyn ES (60')<br><br><u>Total: 80'</u>

Data analysis

All dynamic scan data will be corrected for decay, scatter, randoms and (measured) photon attenuation and be reconstructed as 256 x 256 matrices. A spatial resolution of less than 3 mm full width at half maximum (FWHM) on the complete field of view (FOV) will be achieved. PET and MRI scans will be co-registered and regions of interest (ROI) will be defined on anatomical structures outlined within the MRI scan. These ROI will be projected onto the $[^{15}O]H_2O$ and $[^{18}F]$FDG scans, thus creating tissue-time activity curves. CBF and CMR_{glu} and glucose extraction fraction will be derived for these ROI using previously described methods [60, 96, 97]. In addition, CBF and CMR_{glu} will be calculated on a voxel-by-voxel basis using a basis function and a Patlak approach, respectively [98-102].

5.3 Protocol structural and functional MRI

After 12 weeks of treatment, patients will visit the neuro-imaging centre in the early morning; they will be instructed to be in a fasting state and not to inject their prandial insulin. All scans will be acquired at the same time of the day to minimise diurnal variations. Patients will be made comfortable in a supine position on the scanner table. If they are using glasses, they will receive a special pair that can be used in the MRI-scannner.

Blood glucose values should be maintained at values ranging from 5-10 mmol/l. Therefore, blood glucose values will be checked (and adjusted, if necessary, by the infusion of 5% glucose) before and after testing using a finger prick.

Imaging will be performed on a General Electric (GE) Signa 3T scanner using a phased-array head coil, with foam padding to restrict head motion. A localizer scan will first be performed for positioning of the image planes, followed by an automated shim procedure to improve magnetic field homogeneity.

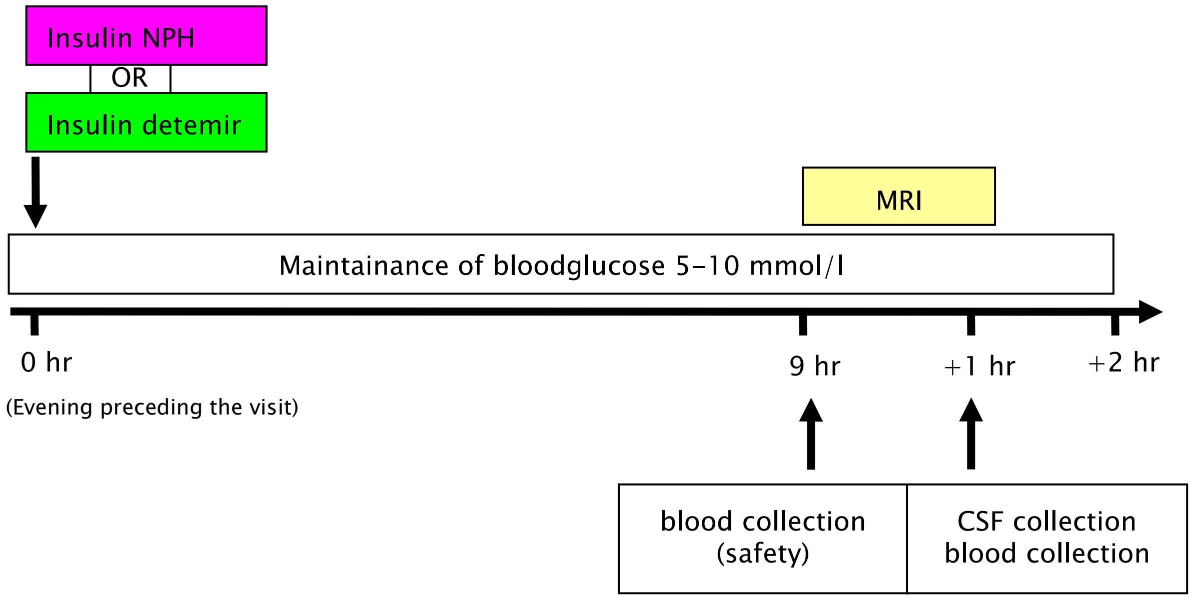

Structural MRI (sMRI)

After either treatment period, the MRI session will end with a functional MRI and multi-sliced pulsed ASL (arterial spin labelling), to measure cerebral perfusion. To limit total acquisition time, and because brain structure is not expected to change over 12 weeks of treatment, the structural MRI sequences will be split and obtained on the two separate scanning days (at week 12 and at week 24). At week 12 (= after the first treatment period), the MRI-session will start with a 3D- T1- weighted MPRAGE (magnetization prepared rapid acquisition gradient echo). At week 24 (= after the second

treatment period), 3D-T2 and 3D-FLAIR (fluid attenuating inverse recovery), will be obtained, to identify white matter lesions.

Functional MRI (fMRI)
During the fasting state, the effects of food-related and non-food-related stimuli on brain activation, and the effect of fasting on cognitive performance (memory), will be investigated, comparing both study insulins. Patients will be instructed about the scanning procedure, so they know what to expect. They will however not be aware of the food-related nature of the experiment prior to viewing of the stimuli in the scanner.
The fMRI will start with a resting state (RS) measurement. The following study design will be used [103-106]; in short, a series of food-related as well as non-food-related visual stimuli will be presented via a computer and back-projected onto a translucent screen, visible for the patients via a mirror. A two-button system will be applied to the patients' right hand. The volunteers will be asked to press the left button when the picture is taken "inside" or the right button when it is taken "outside"; this is to test the level of concentration, and to test whether this depends on the nature of the pictures (food-related versus non-food-related) that are shown. After this 'encoding' part of the experiment, ASL measurements will be made, which will take about 8 minutes. Then, a second series of pictures will be shown, using all pictures of the first series mixed with the same number of new pictures. The volunteers will be asked to press the left button when a new picture is seen or the right button when an old picture is recognised ('retrieval' part of the experiment). Correct answers will not be given.

Data analysis
MRI data will be analysed using Statistical Parametric Mapping (SPM2) software, developed by the Wellcome Dept. of Cognitive Neurology, London, UK.

Summary MRI protocol (total scanning time: 44 min):
1. 3D T1 weighted MPRAGE (week 12) OR
   3D FLAIR and 3D T2 to identify white matter changes (week 24)
2. fMRI resting state
3. fMRI encoding
4. Multi-slice pulsed ASL to measure cerebral perfusion
5. fMRI retrieval

Treatment 12 w		Treatment 12 w	
Detemir		NPH	
NPH		Detemir	
	<u>MRI</u><br>T1<br><br>fMRI RS<br>fMRI encoding<br>ASL<br>fMRI retrieval<br><u>Total: 43’</u>		<u>MRI</u><br>FLAIR<br>T2<br>fMRI RS<br>fMRI encoding<br>ASL<br>fMRI retrieval<br><u>Total: 44’</u>

5.4 Psychological assessment

The following questionnaires will be used:

- The Dutch version of the Eating Disorder Inventory (EDI-II) [107], to screen for eating disorders (visit 1).
- The Dutch version of the Center for Epidemiologic Studies Depression (CES-D) scale, one of the most widely used self-report instruments to measure current depressive symptomatology and to identify possible cases of depressive disorders [115-118] (visit 1).
- The Dutch Eating Behaviour Questionnaire [108-110]. This scale consists of 33 items and contains three scales for the measurement of emotional eating (overeating in response to emotions, 13 items), externally induced eating (eating in response to food related stimuli, regardless of the internal states of hunger and satiety, 10 items) and restrained eating (attempts to refrain from eating, 10 items) All items are rated on a five point scale ranging from 1 (seldom) to 5 (very often) and by dividing the total score by the number of valid answers, the overall score per type of eating behaviour can be calculated (range 1–5). Internal reliability coefficients (Cronbach’s α) are 0,92 (0,93 in obese and 0,92 in non-obese) for the emotional eating scale, 0,80 (0,82 and 0,79 respectively) for the external eating scale and 0,93 (0,93 and 0,92 respectively) for the restraint scale in a sample of obese and non-obese men (45) (visit 2, 3, 5).
- 10-point Likert scale, to measure state of appetite, including hunger, fullness, desire to eat and prospective consumption [111, 112] (visit 3, 4, 5, 6).

- The Dutch version of the shortened Profile of Mood States (POMS) [113, 114], to assess current mood. The POMS is a self-report questionnaire consisting of 32 adjectives describing mood states which will be scored between 0 to 4. The described mood states are depression, anger, fatigue, vigor and tension (visit 3, 4, 5, 6).

6. STATISTICAL CONSIDERATIONS

6.1 General considerations

The null hypotheses will be tested at a two-sided level of 0.05. The null hypothesis *H0: No difference exists after a 12-week treatment with insulin detemir compared to NPH insulin with respect to the parameter of interest*. The null hypothesis will be tested against the hypothesis *H1: A difference exists after a 12-week treatment with insulin detemir compared to NPH insulin with respect to parameter of interest.* Last observation carried forward (LOCF) method will be used to impute missing data if necessary, however, baseline values will not be carried forward. A detailed statistical analysis plan will be produced prior to conduct of final data analysis.

6.2 Analysis population

The intention to treat (ITT) population will consist of all subjects who took at least one dose of the randomized trial insulin beginning at the day of randomization. The evaluable population will consist of all ITT subjects who complete both 12-week treatment periods in compliance with the protocol.

6.3 Primary study endpoints

As described before, the primary study endpoints of this study are:

I. Cerebral metabolic rate of glucose (CMR_{glu}) expressed as umol/100mg/min, in brain regions associated with appetite regulation (e.g. amygdala, orbitofrontal cortex, insula, hypothalamus), as determined by FDG-PET scanning

II. Cerebral blood flow (CBF), expressed as ml/100mg/min, in brain regions associated with appetite regulation (e.g. amygdala, orbitofrontal cortex, insula, hypothalamus), as determined by H2O-PET scanning

6.4 Justification of sample size

Sample size calculation is based on the primary endpoint, as determined by FDG- and H2O-PET.
It is yet unknown whether any differences exist between insulin detemir and NPH insulin on these primary endpoints, since these have not been investigated before; therefore a minimal clinically relevant difference can only be estimated. No differential carry-over effect is expected [10, 119], so the study will be powered based on the cross-over design.

Based on the assumption that the standard deviation is 10% for CMR_{glu} and 20% for CBF - physiological and methodological variability (personal experience; [58, 97, 120, 121])- a total of 32 patients will be needed in this crossover study. With this number of patients we are able (with a power of 80% and a two sided 5% significance level) to detect a difference of 7,2% in glucose consumption and 14,5% in perfusion between both treatment regimens. To account for a drop-out rate of 25%, a total of 40 patients will be included.

6.5 Data analysis

Statistical analysis will be performed using the most recent version of SPSS (SPSS, Chicago, IL). PET and MRI scans will be co-registered and regions of interest (ROI) will be defined on anatomical structures outlined within the MRI scan. These ROI will be projected onto the $[^{15}O]H_2O$ and $[^{18}F]$FDG scans, thus creating tissue-time activity curves. CBF and CMR_{glu} and glucose extraction fraction will be derived for these ROI using previously described methods [60, 96, 97]. In addition, CBF and CMR_{glu} will be calculated on a voxel-by-voxel basis using a basis function and a Patlak approach, respectively [98-102]. These functional images will be used for an analysis using SPM (statistical parametric mapping). The effects on the main and secondary outcomes will be analysed by routine tests for parametric and non-parametric comparison (analysis of variance and multiple regression modelling). A p-value of 0.05 will be considered statistically significant. A more detailed statistical analysis plan will be written prior to the completion of data collection.

7. STUDY MEDICATION

7.1 Storage and provision

Insulin detemir (Levemir®), NPH insulin (Insulatard®) and insulin aspart (NovoRapid®) will be stored in the in-hospital-pharmacy. The trial pharmacist will be responsible for ensuring that all study insulin is stored in a refrigerator, at 2-8 C, protected from exposure to any environmental changes and in a locked facility. Only the investigator and the trial pharmacist (’s assistant) will have access to the drug supplies.

7.2 Randomization of study medication

After review of their eligibility and final assessments, subjects will be randomized - by sequential randomisation - to the insulin to start with. The pharmacist will be responsible for randomization in this open-label study.

7.3 Dose titration

Patients will switch from their pre-trial basal insulin to the trial insulin on a unit-to-unit base if possible; when switching from detemir to NPH in case of large quantities, a 10% decrease in dose will be advised. Basal pre-dinner doses will be titrated to achieve target fasting blood glucose levels of 4-7 mmol/l, while avoiding hypoglycaemia. The following algorithm can be used as a guideline (SPC detemir); however, individual adjustments will be made:

Mean self measured PG pre-breakfast	Dose adjustment (Levemir)
> 10,0 mmol/l (180 mg/dl)<br>9,1-10,0 mmol/ (163-180 mg/dl)<br>8,1-9,0 mmol/l (145-162 mg/dl)<br>7,1-8,0 mmol/l (127-144 mg/dl)<br>6,1-7,0 mmol/l (109-126 mg/dl)	+ 8<br>+ 6<br>+ 4<br>+ 2<br>+ 2
When self-measured PG is (once):	
3,1-4,0 mmol/l (56-72 mg/dl)<br>< 3,1 mmol/l (< 56 mg/dl)	– 2<br>– 4

Patients are instructed carefully how to adjust their insulin dose and how to report their bloodglucose values to the research physician (by phone/ email). After dose adjustment, a nightly plasma glucose control at 3:00 am is advised. Dosing targets are not to be approached at the expense of causing problematic hypoglycaemia. The insulin aspart dose is initially not to be changed during the titration period, if possible. Titration is aimed to achieve post-meal glucoses of < 9 mmol/l. According to the investigator's judgement, doses can be individually adjusted.

8. STUDY DRUG DISCONTINUATION / WITHDRAWAL FROM THE STUDY

Study drug must be discontinued for a given patient if the investigator determines that continuing it would result in a significant safety risk for that patient. After the patient has entered the study, his treatment will be discontinued if one of or more of the following pertain(s):

- The occurrence of an adverse event or clinically significant laboratory change or abnormality that, in the judgment of the investigator, warrants discontinuation of treatment
- Patient's own decision
- Subjects need medical procedures that are not allowed in the protocol
- Subjects cannot undergo the procedures to investigate the research questions as outlined in this protocol

- Non-compliance
- In addition to these requirements for study drug discontinuation, the investigator should discontinue study drug for a given patient if, on balance, he thinks that continuation would be detrimental to the patient's well-being.

The physician can be guided by the criteria above, but may discontinue patients at any time based on her clinical judgment.

9. STUDY COMPLETION

The study will be considered completed for an individual patient when he completes both treatment phases, followed by the planned procedures. The study as a whole will be considered completed when all planned randomized patients have completed both treatment phases, followed by the final measurements.

10. LABORATORY PROCEDURES / TOTAL BLOOD VOLUME

Laboratory procedures will be performed at VUMC laboratories (Department of Clinical Chemistry and Endocrinology) and partly at Novo Nordisk A/S. Blood will be drawn during visit 1, 2, 3, 4, 5 and 6; for an overview see Appendix 1. During the study, 2 PET scans will be obtained, during which a maximum of 2x 250= 500 ml of blood will be drawn.

Maximal estimated blood volume to be obtained from 1 subject during the 24-week study period:

Screening (visit 1)	approx.	10 ml
Visit 2		3 ml
Visit 4 & 6 : 2 x max. 200		400 ml
PET : 2x 250		500 ml
Total blood volume		913 ml

11. INFORMED CONSENT FORM FOR TRIAL SUBJECTS

Prior to any trial related activity, the investigator will give oral and written information about the trial in a form that the subject can read and understand. The investigator must ensure the subject is fully informed about the aims of the trial, procedures, potential risks, any discomforts and expected benefits. The investigator must ensure the subject is informed and agrees that VUmc personnel, their representatives and possibly health authority (national or other) personnel can require access to the patient's data. It must be emphasized that participation is voluntary and that the patient has the right to withdraw from the trial at any time without prejudice. A voluntary, signed and dated Informed Consent will be obtained from the subject prior to any trial related procedure. The written informed

consent must be signed by the person who conducted the informed consent. In obtaining and documenting informed consent, the investigator must comply with the applicable local regulatory requirements and adhere to the ICH GCP guideline and the Declaration of Helsinki.
If information becomes available that may be relevant to the subject's willingness to continue participating in the trial, the investigator must inform the subject in a timely manner, and a revised written informed consent must be obtained.
Unexpected findings will be reported to the participant and to his general practitioner. If the participant does not want to be informed, he/she cannot participate in the trial.
A physician (Prof. dr. Y.M. Smulders, internal medicine, Tel: 020-4444307) who is not involved nor has any interest in the performance of the trial will be available; this person is knowledged about the protocol and can be consulted by subjects potentially interested in participation.

12. FINANCIAL COMPENSATION

All subjects will receive a financial compensation for their participation and reimbursement for travelling expenses will be given based on prices of public transportation.

13. ETHICS

The trial will be conducted in accordance with the Declaration of Helsinki for biomedical research involving human subjects and in accordance with the ICH guidelines for Good Clinical Practice.

14. PUBLICATION POLICY

The investigator has the right to publish (including abstracts) the entire results of the study. Any scientific paper, presentation, communication or other information concerning this project must be submitted in writing to Novo Nordisk prior to submission for publication/presentation for comments. Novo Nordisk will respond within four weeks. All Intellectual Property created and provided by the Sponsor (Novo Nordisk) shall remain the sole property of the Sponsor. The Principal Investigator shall promptly disclose and assign to the Sponsor all inventions and discoveries made by the Principal Investigator related to the Trial. The Principal Investigator shall have a royalty-free right to use the results for non-commercial research and teaching purposes. Novo Nordisk shall have two weeks after receipt of any proposed publication, to object to such publication (or any communication) because there is patentable subject matter which needs protection and/or there is proprietary information of Novo Nordisk contained in the proposed publication. In the event of Novo Nordisk making an objection, the investigator shall refrain from making such publication for a maximum of three months from the date of receipt of the publication in order for Novo Nordisk to file patent application(s) with the applicable patent office(s).

The study will be the main part of a PhD project for one PhD student. A PhD-project has to fulfill the requirements of the Medical Schools at VU University, Amsterdam. In general, a PhD-thesis has to contain at least 5 chapters, consisting of manuscripts published in peer reviewed international journals. We expect that this study will yield sufficient data to meet these criteria.
Data will be presented at relevant national and international scientific meetings, including those from the Dutch Society for Diabetes Research, American Diabetes Association, European Association for Diabetes Research etc. Papers reporting data from the present study will be submitted to appropriate international scientific journals (depending on the results), including Diabetes, Diabetes Care, Diabetologia, Metabolism, Neurology etc.

15. INSURANCE

In case of possible damage as a result of participating in this research project, the loss is, in agreement with legal demands, covered by insurance. The address of the insurance company is:

Onderlinge Waarborgmaatschappij Centramed b.a.
Postbus 191
2270 AD Voorburg
The Netherlands

In Dutch:
Ingevolge art. 7 van de Wet medisch -wetenschappelijk onderzoek met mensen (Staatsblad 1998, 161) is door de verrichter van het onderzoek, het VUmc, een verzekering afgesloten die de door het onderzoek veroorzaakte schade door dood of letsel van de deelnemende proefpersonen dekt. Deze verzekering is afgesloten bij Onderlinge Waarborgmaatschappij Centramed b.a. , Postbus 191, 2270 AD Voorburg. De verzekeraar en de verzekering voldoen aan het besluit verplichte verzekering bij medisch-wetenschappelijk met mensen (Staatsblad 2003, 266) gestelde eisen. Aan het onderzoek deelnemende proefpersonen zullen schriftelijk worden geïnformeerd over de verzekering.

16. ABBREVIATIONS

AD: Alzheimer’s disease, AE: adverse event; AF: alkalic fosfatase, AGE: advanced glycation endproduct, ALAT: alaninie-aminotransferase, ALE: advanced lipoxidation endproduct, ASAT: aspartaat-aminotransferase, BMI: body mass index, CBF: cerebral blood flow, CEL: N^{ε}-(carboxyethyl)-lysine, CML: N^{ε}-(carboxymethyl)-lysine, CMR: cerebral metabolic rate, CNS: central nerve system, CSF: cerebrospinal fluid, 3DG: 3-deoxyglucosone, DIR: double inversion recovery, DM: diabetes mellitus, DRP: diabetic retinopathy, DSP: diabetic sensorimotor polyneuropathy, FDG: fluorodeoxyglucose, FLAIR: fluid attenuating inverse recovery, fMRI: functional MRI; FOV: field of view; FWHM: full width half maximum; γ-GT: γ - glutamyl transferase, IL: interleukine, LAREB: Landelijke Registratie en Evaluatie Bijwerkingen (Netherlands Pharmacovigilance Centre); MBL: mannose-binding lectine; MCI: mild cognitive impairment, MDMA: 3,4-methyleendioxymethamphetamine, MPRAGE: magnetization prepared rapid aquisition gradient echo, MRI: magnetic resonance imaging, NPH: neutral protamine hagedorn, PET: positron emission tomography, ROI: region of interest, SAE: serious adverse event; SPM: statistical parametric mapping, SUSAR: suspected unexpected serious adverse reaction; TNFalpha: tumor necrosis factor alpha.

17. References

1. *The effect of intensive treatment of diabetes on the development and progression of long-term complications in insulin-dependent diabetes mellitus. The Diabetes Control and Complications Trial Research Group.* N Engl J Med, 1993. **329**(14): p. 977-86.
2. *Hypoglycemia in the Diabetes Control and Complications Trial. The Diabetes Control and Complications Trial Research Group.* Diabetes, 1997. **46**(2): p. 271-86.
3. *Weight gain associated with intensive therapy in the diabetes control and complications trial. The DCCT Research Group.* Diabetes Care, 1988. **11**(7): p. 567-73.
4. *Influence of intensive diabetes treatment on body weight and composition of adults with type 1 diabetes in the Diabetes Control and Complications Trial.* Diabetes Care, 2001. **24**(10): p. 1711-21.
5. Carlson, M.G. and P.J. Campbell, *Intensive insulin therapy and weight gain in IDDM.* Diabetes, 1993. **42**(12): p. 1700-7.
6. Wing, R.R., R. Klein, and S.E. Moss, *Weight gain associated with improved glycemic control in population-based sample of subjects with type I diabetes.* Diabetes Care, 1990. **13**(11): p. 1106-9.
7. *Intensive blood-glucose control with sulphonylureas or insulin compared with conventional treatment and risk of complications in patients with type 2 diabetes (UKPDS 33). UK Prospective Diabetes Study (UKPDS) Group.* Lancet, 1998. **352**(9131): p. 837-53.
8. Makimattila, S., K. Nikkila, and H. Yki-Jarvinen, *Causes of weight gain during insulin therapy with and without metformin in patients with Type II diabetes mellitus.* Diabetologia, 1999. **42**(4): p. 406-12.
9. Havelund, S., et al., *The mechanism of protraction of insulin detemir, a long-acting, acylated analog of human insulin.* Pharm Res, 2004. **21**(8): p. 1498-504.
10. Hermansen, K., et al., *Comparison of the soluble basal insulin analog insulin detemir with NPH insulin: a randomized open crossover trial in type 1 diabetic subjects on basal-bolus therapy.* Diabetes Care, 2001. **24**(2): p. 296-301.
11. Heise, T., et al., *Lower within-subject variability of insulin detemir in comparison to NPH insulin and insulin glargine in people with type 1 diabetes.* Diabetes, 2004. **53**(6): p. 1614-20.
12. Plank, J., et al., *A double-blind, randomized, dose-response study investigating the pharmacodynamic and pharmacokinetic properties of the long-acting insulin analog detemir.* Diabetes Care, 2005. **28**(5): p. 1107-12.
13. Danne, T., et al., *Insulin detemir is characterized by a consistent pharmacokinetic profile across age-groups in children, adolescents, and adults with type 1 diabetes.* Diabetes Care, 2003. **26**(11): p. 3087-92.
14. Heinemann, L., Sinha, K., Weyer, C., Loftager, M., Hirschberger, S., Heise, T., *Time-action profileof the soluble, fatty acid acetylated, long-acting insulin analogue NN304.* Diabet Med, 1999. **16**: p. 332-338.
15. De Leeuw, I., et al., *Insulin detemir used in basal-bolus therapy in people with type 1 diabetes is associated with a lower risk of nocturnal hypoglycaemia and less weight gain over 12 months in comparison to NPH insulin.* Diabetes Obes Metab, 2005. **7**(1): p. 73-82.
16. Hermansen, K., et al., *Insulin analogues (insulin detemir and insulin aspart) versus traditional human insulins (NPH insulin and regular human insulin) in basal-bolus therapy for patients with type 1 diabetes.* Diabetologia, 2004. **47**(4): p. 622-9.

17. Home, P., et al., *Insulin detemir offers improved glycemic control compared with NPH insulin in people with type 1 diabetes: a randomized clinical trial.* Diabetes Care, 2004. **27**(5): p. 1081-7.
18. Russell-Jones, D., et al., *Effects of QD insulin detemir or neutral protamine Hagedorn on blood glucose control in patients with type I diabetes mellitus using a basal-bolus regimen.* Clin Ther, 2004. **26**(5): p. 724-36.
19. Vague, P., et al., *Insulin detemir is associated with more predictable glycemic control and reduced risk of hypoglycemia than NPH insulin in patients with type 1 diabetes on a basal-bolus regimen with premeal insulin aspart.* Diabetes Care, 2003. **26**(3): p. 590-6.
20. Haak, T., et al., *Lower within-subject variability of fasting blood glucose and reduced weight gain with insulin detemir compared to NPH insulin in patients with type 2 diabetes.* Diabetes Obes Metab, 2005. **7**(1): p. 56-64.
21. Pieber, T.R., et al., *Comparison of three multiple injection regimens for Type 1 diabetes: morning plus dinner or bedtime administration of insulin detemir vs. morning plus bedtime NPH insulin.* Diabet Med, 2005. **22**(7): p. 850-7.
22. Standl, E., H. Lang, and A. Roberts, *The 12-month efficacy and safety of insulin detemir and NPH insulin in basal-bolus therapy for the treatment of type 1 diabetes.* Diabetes Technol Ther, 2004. **6**(5): p. 579-88.
23. Philis-Tsimikas, A., et al., *Comparison of once-daily insulin detemir with NPH insulin added to a regimen of oral antidiabetic drugs in poorly controlled type 2 diabetes.* Clin Ther, 2006. **28**(10): p. 1569-81.
24. Hordern, S.V., et al., *Comparison of the effects on glucose and lipid metabolism of equipotent doses of insulin detemir and NPH insulin with a 16-h euglycaemic clamp.* Diabetologia, 2005. **48**(3): p. 420-6.
25. Home, P. and P. Kurtzhals, *Insulin detemir: from concept to clinical experience.* Expert Opin Pharmacother, 2006. **7**(3): p. 325-43.
26. Hordern, S.V. and D.L. Russell-Jones, *Insulin detemir, does a new century bring a better basal insulin?* Int J Clin Pract, 2005. **59**(6): p. 730-9.
27. Hermansen, K. and M. Davies, *Does insulin detemir have a role in reducing risk of insulin-associated weight gain?* Diabetes Obes Metab, 2007. **9**(3): p. 209-17.
28. Riddle, M.C., J. Rosenstock, and J. Gerich, *The treat-to-target trial: randomized addition of glargine or human NPH insulin to oral therapy of type 2 diabetic patients.* Diabetes Care, 2003. **26**(11): p. 3080-6.
29. Hermansen, K., et al., *A 26-week, randomized, parallel, treat-to-target trial comparing insulin detemir with NPH insulin as add-on therapy to oral glucose-lowering drugs in insulin-naive people with type 2 diabetes.* Diabetes Care, 2006. **29**(6): p. 1269-74.
30. Hopkins, D.F. and G. Williams, *Insulin receptors are widely distributed in human brain and bind human and porcine insulin with equal affinity.* Diabet Med, 1997. **14**(12): p. 1044-50.
31. Schulingkamp, R.J., et al., *Insulin receptors and insulin action in the brain: review and clinical implications.* Neurosci Biobehav Rev, 2000. **24**(8): p. 855-72.
32. Hennige, A.M., et al., *Tissue selectivity of insulin detemir action in vivo.* Diabetologia, 2006. **49**(6): p. 1274-82.
33. Kern, W., et al., *Central nervous system effects of intranasally administered insulin during euglycemia in men.* Diabetes, 1999. **48**(3): p. 557-63.

34. Born, J., et al., *Sniffing neuropeptides: a transnasal approach to the human brain.* Nat Neurosci, 2002. **5**(6): p. 514-6.
35. Hallschmid, M., et al., *Intranasal insulin reduces body fat in men but not in women.* Diabetes, 2004. **53**(11): p. 3024-9.
36. Hallschmid, M., et al., *Obese men respond to cognitive but not to catabolic brain insulin signaling.* Int J Obes (Lond), 2007.
37. Tschritter, O., et al., *The cerebrocortical response to hyperinsulinemia is reduced in overweight humans: a magnetoencephalographic study.* Proc Natl Acad Sci U S A, 2006. **103**(32): p. 12103-8.
38. Anthony, K., et al., *Attenuation of insulin-evoked responses in brain networks controlling appetite and reward in insulin resistance: the cerebral basis for impaired control of food intake in metabolic syndrome?* Diabetes, 2006. **55**(11): p. 2986-92.
39. Kern, W., et al., *Low cerebrospinal fluid insulin levels in obese humans.* Diabetologia, 2006. **49**(11): p. 2790-2.
40. Morris, J.S. and R.J. Dolan, *Involvement of human amygdala and orbitofrontal cortex in hunger-enhanced memory for food stimuli.* J Neurosci, 2001. **21**(14): p. 5304-10.
41. Tataranni, P.A., et al., *Neuroanatomical correlates of hunger and satiation in humans using positron emission tomography.* Proc Natl Acad Sci U S A, 1999. **96**(8): p. 4569-74.
42. Small, D.M., et al., *Changes in brain activity related to eating chocolate: from pleasure to aversion.* Brain, 2001. **124**(Pt 9): p. 1720-33.
43. Benedict, C., et al., *Intranasal insulin improves memory in humans.* Psychoneuroendocrinology, 2004. **29**(10): p. 1326-34.
44. Debons, A.F., I. Krimsky, and A. From, *A direct action of insulin on the hypothalamic satiety center.* Am J Physiol, 1970. **219**(4): p. 938-43.
45. Rodin, J., et al., *Effect of insulin and glucose on feeding behavior.* Metabolism, 1985. **34**(9): p. 826-31.
46. Plum, L., B.F. Belgardt, and J.C. Bruning, *Central insulin action in energy and glucose homeostasis.* J Clin Invest, 2006. **116**(7): p. 1761-6.
47. Obici, S., et al., *Decreasing hypothalamic insulin receptors causes hyperphagia and insulin resistance in rats.* Nat Neurosci, 2002. **5**(6): p. 566-72.
48. Obici, S., et al., *Hypothalamic insulin signaling is required for inhibition of glucose production.* Nat Med, 2002. **8**(12): p. 1376-82.
49. Banks, W.A., et al., *Transport of insulin across the blood-brain barrier: saturability at euglycemic doses of insulin.* Peptides, 1997. **18**(9): p. 1423-9.
50. Schwartz, M.W., *Brain pathways controlling food intake and body weight.* Exp Biol Med (Maywood), 2001. **226**(11): p. 978-81.
51. Schwartz, M.W., *Central nervous system regulation of food intake.* Obesity (Silver Spring), 2006. **14 Suppl 1**: p. 1S-8S.
52. Schwartz, M.W., et al., *Central nervous system control of food intake.* Nature, 2000. **404**(6778): p. 661-71.
53. Woods, S.C., et al., *Signals that regulate food intake and energy homeostasis.* Science, 1998. **280**(5368): p. 1378-83.
54. Diamant, M., *Brain insulin signalling in the regulation of energy balance and peripheral metabolism.* Ideggyogy Sz, 2007. **60**(3-4): p. 97-108.
55. Kern, W., et al., *Effects of insulin and hypoglycemia on the auditory brain stem response in humans.* J Neurophysiol, 1994. **72**(2): p. 678-83.

56. Hasselbalch, S.G., et al., *No effect of insulin on glucose blood-brain barrier transport and cerebral metabolism in humans.* Diabetes, 1999. **48**(10): p. 1915-21.
57. Shapiro, E.T., et al., *Change in hexose distribution volume and fractional utilization of [18F]-2-deoxy-2-fluoro-D-glucose in brain during acute hypoglycemia in humans.* Diabetes, 1990. **39**(2): p. 175-80.
58. Cranston, I., et al., *Regional differences in cerebral blood flow and glucose utilization in diabetic man: the effect of insulin.* J Cereb Blood Flow Metab, 1998. **18**(2): p. 130-40.
59. Seaquist, E.R., et al., *The effect of insulin on in vivo cerebral glucose concentrations and rates of glucose transport/metabolism in humans.* Diabetes, 2001. **50**(10): p. 2203-9.
60. Bingham, E.M., et al., *The role of insulin in human brain glucose metabolism: an 18fluoro-deoxyglucose positron emission tomography study.* Diabetes, 2002. **51**(12): p. 3384-90.
61. Taylor, V.H. and G.M. MacQueen, *Cognitive dysfunction associated with metabolic syndrome.* Obes Rev, 2007. **8**(5): p. 409-18.
62. Bar, K.J., et al., *Pentosidine and N(epsilon)-(carboxymethyl)-lysine in Alzheimer's disease and vascular dementia.* Neurobiol Aging, 2003. **24**(2): p. 333-8.
63. Ahmed, N., et al., *Protein glycation, oxidation and nitration adduct residues and free adducts of cerebrospinal fluid in Alzheimer's disease and link to cognitive impairment.* J Neurochem, 2005. **92**(2): p. 255-63.
64. Sell, D.R., et al., *Pentosidine formation in skin correlates with severity of complications in individuals with long-standing IDDM.* Diabetes, 1992. **41**(10): p. 1286-92.
65. McCance, D.R., et al., *Maillard reaction products and their relation to complications in insulin-dependent diabetes mellitus.* J Clin Invest, 1993. **91**(6): p. 2470-8.
66. Beisswenger, P.J., et al., *Formation of immunochemical advanced glycosylation end products precedes and correlates with early manifestations of renal and retinal disease in diabetes.* Diabetes, 1995. **44**(7): p. 824-9.
67. Vlassara, H., R. Bucala, and L. Striker, *Pathogenic effects of advanced glycosylation: biochemical, biologic, and clinical implications for diabetes and aging.* Lab Invest, 1994. **70**(2): p. 138-51.
68. Jakus, V., et al., *Values of markers of early and advanced glycation and lipoxidation in serum proteins of children with diabetes mellitus.* Bratisl Lek Listy, 2000. **101**(9): p. 484-9.
69. Jakus, V., et al., *Serum levels of advanced glycation end products in poorly metabolically controlled children with diabetes mellitus: relation to HbA1c.* Diabetes Nutr Metab, 2001. **14**(4): p. 207-11.
70. Jakus, V. and N. Rietbrock, *Advanced glycation end-products and the progress of diabetic vascular complications.* Physiol Res, 2004. **53**(2): p. 131-42.
71. Craft, S., *Insulin resistance syndrome and Alzheimer's disease: age- and obesity-related effects on memory, amyloid, and inflammation.* Neurobiol Aging, 2005. **26 Suppl 1**: p. 65-9.
72. Watson, G.S., et al., *Insulin increases CSF Abeta42 levels in normal older adults.* Neurology, 2003. **60**(12): p. 1899-903.
73. Elliott, E. and I. Ginzburg, *The role of neurotrophins and insulin on tau pathology in Alzheimer's disease.* Rev Neurosci, 2006. **17**(6): p. 635-42.

74. Benedict, C., et al., *Intranasal insulin to improve memory function in humans.* Neuroendocrinology, 2007. **86**(2): p. 136-42.
75. Kern, W., et al., *Improving influence of insulin on cognitive functions in humans.* Neuroendocrinology, 2001. **74**(4): p. 270-80.
76. Craft, S., et al., *Insulin dose-response effects on memory and plasma amyloid precursor protein in Alzheimer's disease: interactions with apolipoprotein E genotype.* Psychoneuroendocrinology, 2003. **28**(6): p. 809-22.
77. Ferguson, S.C., et al., *Apolipoprotein-e influences aspects of intellectual ability in type 1 diabetes.* Diabetes, 2003. **52**(1): p. 145-8.
78. Wilson, R.S., et al., *The apolipoprotein E epsilon 2 allele and decline in episodic memory.* J Neurol Neurosurg Psychiatry, 2002. **73**(6): p. 672-7.
79. Bennet, A.M., et al., *Association of apolipoprotein E genotypes with lipid levels and coronary risk.* Jama, 2007. **298**(11): p. 1300-11.
80. Matsuda, M., et al., *Altered hypothalamic function in response to glucose ingestion in obese humans.* Diabetes, 1999. **48**(9): p. 1801-6.
81. Gautier, J.F., et al., *Effect of satiation on brain activity in obese and lean women.* Obes Res, 2001. **9**(11): p. 676-84.
82. Gautier, J.F., et al., *Differential brain responses to satiation in obese and lean men.* Diabetes, 2000. **49**(5): p. 838-46.
83. Del Parigi, A., et al., *Neuroimaging and obesity: mapping the brain responses to hunger and satiation in humans using positron emission tomography.* Ann N Y Acad Sci, 2002. **967**: p. 389-97.
84. Karhunen, L.J., et al., *Regional cerebral blood flow during food exposure in obese and normal-weight women.* Brain, 1997. **120 (Pt 9)**: p. 1675-84.
85. Kaiyala, K.J., et al., *Obesity induced by a high-fat diet is associated with reduced brain insulin transport in dogs.* Diabetes, 2000. **49**(9): p. 1525-33.
86. Tschritter, O., et al., *Cerebrocortical Beta activity in overweight humans responds to insulin detemir.* PLoS ONE, 2007. **2**(11): p. e1196.
87. Clegg, D.J., et al., *Differential sensitivity to central leptin and insulin in male and female rats.* Diabetes, 2003. **52**(3): p. 682-7.
88. Ewing, D.J., et al., *The value of cardiovascular autonomic function tests: 10 years experience in diabetes.* Diabetes Care, 1985. **8**(5): p. 491-8.
89. Bril, V.P., B.A., *Validation of the Toronto Clinical Scoring System for Diabetic Polyneuropathy.* Diabetes Care, 2002. **25**: p. 2048–2052.
90. Aldington, S.J., et al., *Methodology for retinal photography and assessment of diabetic retinopathy: the EURODIAB IDDM complications study.* Diabetologia, 1995. **38**(4): p. 437-44.
91. Langan, S.J., et al., *Cumulative cognitive impairment following recurrent severe hypoglycaemia in adult patients with insulin-treated diabetes mellitus.* Diabetologia, 1991. **34**(5): p. 337-44.
92. Deary, I.J., et al., *Severe hypoglycemia and intelligence in adult patients with insulin-treated diabetes.* Diabetes, 1993. **42**(2): p. 341-4.
93. *Adverse events and their association with treatment regimens in the diabetes control and complications trial.* Diabetes Care, 1995. **18**(11): p. 1415-27.
94. Straus, S.E., K.E. Thorpe, and J. Holroyd-Leduc, *How do I perform a lumbar puncture and analyze the results to diagnose bacterial meningitis?* Jama, 2006. **296**(16): p. 2012-22.

95. de Jong, H.W., et al., *Performance evaluation of the ECAT HRRT: an LSO-LYSO double layer high resolution, high sensitivity scanner.* Phys Med Biol, 2007. **52**(5): p. 1505-26.
96. Studholme, C., D.L. Hill, and D.J. Hawkes, *Automated three-dimensional registration of magnetic resonance and positron emission tomography brain images by multiresolution optimization of voxel similarity measures.* Med Phys, 1997. **24**(1): p. 25-35.
97. Lammertsma, A.A., et al., *Combination of dynamic and integral methods for generating reproducible functional CBF images.* J Cereb Blood Flow Metab, 1990. **10**(5): p. 675-86.
98. Boellaard, R., et al., *Evaluation of basis function and linear least squares methods for generating parametric blood flow images using 15O-water and Positron Emission Tomography.* Mol Imaging Biol, 2005. **7**(4): p. 273-85.
99. Gunn, R.N., et al., *Parametric imaging of ligand-receptor binding in PET using a simplified reference region model.* Neuroimage, 1997. **6**(4): p. 279-87.
100. Patlak, C.S., R.G. Blasberg, and J.D. Fenstermacher, *Graphical evaluation of blood-to-brain transfer constants from multiple-time uptake data.* J Cereb Blood Flow Metab, 1983. **3**(1): p. 1-7.
101. Phelps, M.E., et al., *Tomographic measurement of local cerebral glucose metabolic rate in humans with (F-18)2-fluoro-2-deoxy-D-glucose: validation of method.* Ann Neurol, 1979. **6**(5): p. 371-88.
102. Sokoloff, L., et al., *The [14C]deoxyglucose method for the measurement of local cerebral glucose utilization: theory, procedure, and normal values in the conscious and anesthetized albino rat.* J Neurochem, 1977. **28**(5): p. 897-916.
103. Killgore, W.D., et al., *Cortical and limbic activation during viewing of high- versus low-calorie foods.* Neuroimage, 2003. **19**(4): p. 1381-94.
104. Pelchat, M.L., et al., *Images of desire: food-craving activation during fMRI.* Neuroimage, 2004. **23**(4): p. 1486-93.
105. Killgore, W.D. and D.A. Yurgelun-Todd, *Affect modulates appetite-related brain activity to images of food.* Int J Eat Disord, 2006. **39**(5): p. 357-63.
106. Porubska, K., et al., *Subjective feeling of appetite modulates brain activity: an fMRI study.* Neuroimage, 2006. **32**(3): p. 1273-80.
107. Garner, D.M., *EDI-2: Eating Disorder Inventory-2. Professional manual.* . 1991.
108. Van Strien, T., Frijters, J.E.R., Bergers, G.P.A. & Defares, P.B., *The Dutch Eating Behaviour Questionnaire (DEBQ) for assessment of restrained, emotional and external eating behaviour.* International Journal of Eating Disorders, 1986. **5**: p. 747-755.
109. van de Laar, F.A., et al., *Eating behaviour and adherence to diet in patients with Type 2 diabetes mellitus.* Diabet Med, 2006. **23**(7): p. 788-94.
110. Caccialanza, R., et al., *Validation of the Dutch Eating Behaviour Questionnaire parent version (DEBQ-P) in the Italian population: a screening tool to detect differences in eating behaviour among obese, overweight and normal-weight preadolescents.* Eur J Clin Nutr, 2004. **58**(9): p. 1217-22.
111. Hill, A.J., P.J. Rogers, and J.E. Blundell, *Techniques for the experimental measurement of human eating behaviour and food intake: a practical guide.* Int J Obes Relat Metab Disord, 1995. **19**(6): p. 361-75.
112. Mars, M., et al., *Fasting leptin and appetite responses induced by a 4-day 65%-energy-restricted diet.* Int J Obes (Lond), 2006. **30**(1): p. 122-8.

113. McNair, D., Lorr M, Droppleman LF., *Manual for the Profile of Mood States*. 1971, San Diego CA: Educational and Industrial Testing Service.
114. Wald, F.M., G. J., *De verkorte versie van de Nederlandse vertaling van de Profile of Mood States (POMS).*
The shortened version of the Dutch translation of the Profile of Mood States (POMS). Nederlands Tijdschrift voor de Psychologie en haar Grensgebieden, 1990. **45**: p. 86-90.
115. Bouma, J., Ranchor, A.V., Sanderman, R., Van Sonderen, F.L.P., *Het meten van symptomen van depressie met de CES-D. Een handleiding.* . 1995.
116. Schroevers, M.J., et al., *The evaluation of the Center for Epidemiologic Studies Depression (CES-D) scale: Depressed and Positive Affect in cancer patients and healthy reference subjects.* Qual Life Res, 2000. **9**(9): p. 1015-29.
117. Radloff, L., *The CES-D Scale: A self-reportdepression scale for research in the general population.* Appl. Psychol. Measur., 1977. **1**: p. 385-401.
118. Hanewald, G.J.F.P., *CES-D. De Nederlandse versie. Een onderzoek naar de betrouwbaarheid en de validiteit.* 1987.
119. Brunner, G.A., et al., *Pharmacokinetic and pharmacodynamic properties of long-acting insulin analogue NN304 in comparison to NPH insulin in humans.* Exp Clin Endocrinol Diabetes, 2000. **108**(2): p. 100-5.
120. Frackowiak, R.S., et al., *Quantitative measurement of regional cerebral blood flow and oxygen metabolism in man using 15O and positron emission tomography: theory, procedure, and normal values.* J Comput Assist Tomogr, 1980. **4**(6): p. 727-36.
121. Bartlett, E.J., et al., *Reproducibility of cerebral glucose metabolic measurements in resting human subjects.* J Cereb Blood Flow Metab, 1988. **8**(4): p. 502-12.

Appendix 1: Flow chart

**INcEREBRO**										
**Flow Chart**	**Visits**									
	**1**	**2**	**TC1**	**3**	**4**		**TC2**	**5**	**6**	**E**
	**Screening**	**Randomisation**	**Tel call**	**Measurements**		**Cross-over**	**Tel call**	**Measurements**		**Evaluation**
	Week -4	Week 0	Week 2	Week 12	Week 12		Week 14	Week 24	Week 24	Week 26
		Detemir				NPH				
		NPH				Detemir				
Informed Consent	X									
Incl./Excl. Criteria/ demography	X									
Start of study insulin		X				X				
Insulin Therapy (titration, monitoring hypoglycaemic events etc)	X	X	X	X	X		X	X	X	X
Patient History										
– past	X									
– recent		X	X	X	X		X	X	X	
Physical Examination										
– General	X			X				X		
– DSP	X									
– Ewing test	X									
– Fundoscopy	X									
Vital Signs (incl. body wt)	X	X		X	X			X	X	X
Monitoring (S) AE's			X	X	X		X	X	X	
Concomitant medication	X	X	X	X	X		X	X	X	X
PET-scan				X				X		
fMRI-scan					X				X	
Blood measurements										
– screening	X									
– study parameters		X		X	X			X	X	
Lumbar puncture					X				X	
Questionnaires:										
EDI-II; CES-D	X									
POMS, Likert				X	X			X	X	
DEBQ		X		X				X		
Urine analysis		X		X				X		

Appendix 2: Blood, CSF, urine

	Screen	V2	V3 (PET)	V4 (LP)	V5 (PET)	V6 (LP)
**Blood**						
HbA1c	x	x		x		x
Haemoglobin	x			x		x
Leucocytes/platelets	x			x		x
Creatinine	x			x		x
Albumin			x	x	x	x
Lipid profile	x					
AF, γ-GT	x			x		x
ALT	x			x		x
TSH	x					
Glucose			x	x	x	x
Insulin			x	x	x	x
**Urine**						
Creatinine		x	x		x	
Microalbumine		x	x		x	
**CSF**						
Insulin				x		x
Glucose				x		x
Total cells				x		x
Total protein, albumin				x		x

Appendix 3: Safety considerations and reporting

Introduction

This study is an investigator-initiated trial, which will be performed by investigators of the VU University Medical Center, Amsterdam, The Netherlands. The investigator is responsible for reporting adverse events in accordance with national requirements.

Background

As noted, 40 type1 diabetic patients will be randomly allocated to either insulin detemir or NPH insulin. They will use the trial insulin for a period of 12 weeks, after which they will switch to the other insulin. Participants will be contacted (by phone or email) to ascertain safety and tolerability, as well as compliance.

The ***medication*** used in the study, insulin detemir and NPH insulin, administered subcutaneously, are both registered for the treatment of diabetes mellitus for several years. Participants will be informed about the possible side effects, the most frequent side effect being, as for all insulins, hypoglycaemia. Less frequently occurring side effects are urticaria, rash, diabetic retinopathy, lipodystrophy, locally occurring hypersensitivity reactions, edema. The occurrence of hypoglycaemic episodes will be minimized by repeated instruction and contact with patients by the research physician, close monitoring of blood glucose levels and adjustment of the insulin dosing according to a previously described algorithm.

CSF will be obtained by a ***lumbar puncture***. By using a very thin 25G atraumatic spinal needle, the risk of post-punctional headache is very small; patients will be informed about this potential risk and how to act in case this might happen.

Since ***MRI*** is a non-invasive procedure and no radioactivity is used, participants will not be at any risk during this test. Patients with claustrophobia will not be included in the study.

For ***PET scanning***, an iv-cannula will be inserted for the various infusions and an arterial cannula will be inserted for the assessment of arterial concentrations of metabolites and glucose. A skilled investigator will insert the catheters, in order to minimize the already small risk of infection and bleeding. Due to the infusions, the patients may have the urge to urinate during the investigations. To avoid discomfort, subjects will be asked to urinate before onset of scanning. During scanning, patients will be immobilized in a supine position. The prolonged assumption of the supine position and immobilization during the PET scans may pose some discomfort to the subjects (a total of less than 120 minutes per scan). All effort will be made to minimize discomfort for the patient, within the possibilities of the protocol. To avoid discomfort during scanning, all subjects will be acquainted to the scan area prior to the scans. In case of discomfort, skilled staff will be available to provide support, reduce anxiety, optimize comfort of the subject and remove the subject from the scanner if requested.

Participants will be exposed to a total of 2x (1,2 + 3,52)= 9,44 mSv during the entire study protocol.

15O-H2O 1,2 mSv (1100 MBq)

18FDG 3,52 mSv (185 MBq)

According to the 'Radiological Protection in Biomedical Research' (ICRP) guidelines, which provide justification for biomedical research with ionized radiation, the level of risk related to radiation exposure is minor to intermediate if the effective dose is below or equal to 10 mSv. Exposure of participants to ionized radiation in this project can be justified because this project is in line with the ICRP directive; this project is expected to further increase knowledge concerning the mechanism by which insulin acts *in cerebro*, possibly explaining the observation of detemir being associated with less weight gain, which can have therapeutic consequences for a population already at high risk for obesity and other obesity related complications.

Definitions of - and the procedures for reporting - an Adverse Event (AE)

An AE is defined as any untoward medical occurrence in a patient or clinical investigation subject administered a pharmaceutical product and which does not necessarily have to have a causal relationship with this treatment. An adverse event can therefore be any unfavourable and unintended sign (including an abnormal laboratory finding, for example), symptom, or disease temporally associated with the use of a medicinal product, whether or not considered related to the medicinal product. Detailed information concerning the AE will be collected on specific case report forms and stored in a database and will be reported to LAREB (Landelijke Registratie en Evaluatie Bijwerkingen; Netherlands Pharmacovigilance Centre).

Definitions of - and the procedures for reporting - a Serious Adverse Event (SAE)

A Serious Adverse Event is:

Any untoward medical occurrence that at any dose:

- Results in death
- Is life-threatening
- Requires inpatient hospitalization or prolongation of existing hospitalization
- Results in persistent or significant disability/incapacity
- Is a congenital anomaly/birth defect
- Important medical events that may not result in death, be life-threatening, or require hospitalization may be considered as SAE when, based on appropriate medical judgement, they may jeopardise the subject and may require medical or surgical intervention to prevent one of the outcomes listed in this definition.

Reporting of a SAE

Each serious adverse event will be investigated thoroughly and an assessment will be made whether the SAE is drug-related. Detailed information concerning the SAE will be written down on specific case report forms at the study site. Each re-occurrence, complication or progression of the original event will be written down as a follow-up to that event. The follow-up information should describe whether the event has resolved or continues, if and how it was treated, and whether the patient continued or discontinued study participation. All SAEs will be stored in a database at the study site and reported to the pharmacy, to the Medical Ethics Committee (METC) of the VU University Medical Center and to Novo Nordisk.

In summary, in case of a SAE, the following procedure follows:

- The (principal) investigator is notified within 24 hours
- If a causal relationship with the study (drug) cannot be excluded, the patient is excluded from further trial treatment.
- A detailed documentation of the nature of the SAE is made, as well as of all the interventions taken and of possible effects that might occur in the (near) future. This report is sent to the Medical Ethics Committee and the pharmacy of the local institution as well as to the pharmaceutical company supplying the drug (Novo Nordisk).

Reporting of SUSARS

When the serious adverse event is drug related, and unexpected (since it was not mentioned in the Summary of Product Characteristics), a suspected unexpected serious adverse reaction (SUSAR) has occurred. SUSARS will be reported within 24 hours to METC, CCMO, the involved pharmacy and Novo Nordisk, using specific forms available at CCMO website.

Yearly report

Annually, an overview with study SAEs will be sent to VUmc METC and CCMO.

Contact details

VU University Medical Centre, Department of endocrinology, Amsterdam: drs. L.W. van Golen (research physician) / dr. M. Diamant, PI, (endocrinologist) / dr. M.L. Drent (endocrinologist); phone: +31-20-4440533; fax: +31-20-4440502

VU University Medical Centre, Department of Pharmacy, Amsterdam: dr. P. Bet; phone: +31-20-4443546/ +31-20-4444444/ (tracer *986703)

Novo Nordisk B.V.
t.a.v. Drug Safety Officer
Antwoordnummer 10126
2400 VB ALPHEN aan den RIJN
Phone: +31 (0)172 44 94 94
Fax: +31 (0)172 42 47 09
E-mail: NLAR-DSO@novonordisk.com

Appendix 4: Healthy controls

This INcEREBRO-protocol was designed to investigate whether there are differences between diabetic patients on detemir or NPH insulin in brain regions controlling appetite and satiety – as measured by PET and fMRI –which could explain the differences in weight gain observed in several clinical trials. Because peripheral metabolic signals (a.o. glucose, insulin, free fatty acids, leptin, ghrelin…), known to regulate energy metabolism, satiety and food controlling behaviour via a negative feedback system in the central nervous system are different in diabetic patients as compared to healthy persons, it would be interesting to also compare the studied diabetic patients with age- and BMI-matched healthy controls. Therefore, about 30 healthy volunteers will be included in the protocol. They will have a short screening visit (including informed consent taking, medical history, a physical examination and a screening list to exclude eating disorders, and blood drawing for fasting glucose, liver, renal and thyroid function to make sure they are healthy), after which they will be scheduled for an MRI and a PET scan. Like all patients, all volunteers will be scanned early in the morning in a fasting state. Just before the scans, spare blood will be drawn to measure a.o. glucose, insulin, leptin and ghrelin levels. Before and after scanning, they will fill out the 10-point Likert VAS scale and the POMS mood questionnaire.
